# Genome-Wide Single-Nucleotide Polymorphisms in CMS and Restorer Lines Discovered by Genotyping Using Sequencing and Association with Marker-Combining Ability for 12 Yield-Related Traits in *Oryza sativa* L. subsp. Japonica

**DOI:** 10.3389/fpls.2017.00143

**Published:** 2017-02-08

**Authors:** Imdad U. Zaid, Weijie Tang, Erbao Liu, Sana U. Khan, Hui Wang, Edzesi W. Mawuli, Delin Hong

**Affiliations:** ^1^State Key Laboratory of Crop Genetics and Germplasm Enhancement, Nanjing Agricultural UniversityNanjing, China; ^2^School of Chemistry and Molecular Biosciences, The University of QueenslandBrisbane, QLD, Australia

**Keywords:** japonica hybrid rice, general combining ability, genotyping by sequencing, single nucleotide polymorphisms, SNP-CA associations

## Abstract

Heterosis or hybrid vigor is closely related with general combing ability (GCA) of parents and special combining ability (SCA) of combinations. The evaluation of GCA and SCA facilitate selection of parents and combinations in heterosis breeding. In order to improve combining ability (CA) by molecular marker assist selection, it is necessary to identify marker loci associated with the CA. To identify the single nucleotide polymorphisms (SNP) loci associated with CA in the parental genomes of japonica rice, genome-wide discovered SNP loci were tested for association with the CA of 18 parents for 12 yield-related traits. In this study, 81 hybrids were created and evaluated to calculate the CA of 18 parents. The parents were sequenced by genotyping by sequencing (GBS) method for identification of genome-wide SNPs. The analysis of GBS indicated that the successful mapping of 9.86 × 10^6^ short reads in the Nipponbare reference genome consists of 39,001 SNPs in parental genomes at 11,085 chromosomal positions. The discovered SNPs were non-randomly distributed within and among the 12 chromosomes of rice. Overall, 20.4% (8026) of the discovered SNPs were coding types, and 8.6% (3344) and 9.9% (3951) of the SNPs revealed synonymous and non-synonymous changes, which provide valuable knowledge about the underlying performance of the parents. Furthermore, the associations between SNPs and CA indicated that 362 SNP loci were significantly related to the CA of 12 parental traits. The identified SNP loci of CA in our study were distributed genome wide and caused a positive or negative effect on the CA of traits. For the yield-related traits, such as grain thickness, days to heading, panicle length, grain length and 1000-grain weight, a maximum number of positive SNP loci of CA were found in CMS A171 and in the restorers LC64 and LR27. On an individual basis, some of associated loci that resided on chromosomes 2, 5, 7, 9, and 11 recorded maximum positive values for the CA of traits. From our results, we suggest that heterosis in japonica rice would be improved by pyramiding the favorable SNP loci of CA and eliminating the unfavorable loci from parental genomes.

## Introduction

Hybrid rice breeding has been successfully adopted in 27 rice-growing countries. China is the largest producer and consumer of hybrid rice, and considers itself a pioneer in hybrid rice breeding. With the cultivation of hybrid rice, China obtained 20% more grain yield compared to their best conventional cultivars (Yuan and Virmani, 1988; Cheng et al., 2004). In China, a total of 33 million hectares (ha) of paddy fields are available for rice cultivation, in which 25 million ha are occupied by the indica rice and 8 million ha are occupied by the japonica rice. In the total land of indica rice, 70% are used for indica hybrids rice cultivation, whereas only 5% of the total planted land is used for japonica hybrid cultivations. (Tang et al., 2008). In the past, great achievements were made to increase the growing area and yield productivity of japonica hybrids by improving culturing practices with the use of productive seeds. However, until recently, the yield potential of japonica hybrids has been low and unstable compared to the indica hybrids. Previous breeding practices have confirmed that japonica hybrids encounter several challenges due to their low standard heterosis, which may be caused by their narrow genetic background, their large panicle size with insufficient filling (poor grain plumpness), or the unavailability of elite parental combinations for developing superior hybrids (Xie and Hardy, 2009).

Commercially developed hybrid cultivars exhibit desirable characteristics of yield and quality, that are inherited from their parental lines (Cao and Zhan, 2014). Such parental characteristics are heritable and could be appear in the F_1_ generation in the form of heterosis. The successful selection of proper parents, based on the combining ability of yield-related traits, contributes to yield outcomes in hybrid breeding. The combining ability is a powerful breeding test that estimates the breeding values of parents and crosses in terms of general and specific combinations (Sprague and Tatum, 1942). Conventionally, the combining ability of crossing parents is calculated by evaluating all their developed crosses, which is laborious, tedious and time-consuming (Smith et al., 2008). Moreover, when the number of parents involved in combining ability manipulation become large, their hybrids affect the experimental feasibility (Bertan et al., 2007).

With recent rapid developments in molecular marker technology, it is now feasible to genotype or identify marker loci for CA. Previous studies made this possible and discovered a genetic basis of CA with SSR markers (simple sequence repeats) (Liang et al., 2010; Huang et al., 2013; Liu E. B. et al., 2013; Qi et al., 2013; Liu Y. et al., 2015; Xie et al., 2016). These studies have revealed several SSR marker loci associated with the CA of yield and quality traits. However, such studies have been restricted to SSR markers. To date, no SNP base analysis has been reported for the discovery of SNP locus/loci associated with the CA of parental traits.

Recent advances in next-generation sequencing (NGS) have facilitated genome-wide SNP identification and SNP characterization. The advent of NGS has reduced the cost of genome sequencing to a level, where GBS is now considered a powerful tool for inquiring into a large number of genomic variations (SNPs). The GBS platform is simple, fast and accurate and has been widely practiced for large scale mining of SNPs in many crop species, including wheat (Poland J. et al., 2012), rice (Spindel et al., 2013), soybean (Sonah et al., 2013), chickpea (Kujur et al., 2015), sorghum (Morris et al., 2012) alfalfa (Yu L. X. et al., 2016), and olive (İpek et al., 2016).

SNPs are the most common and abundant sequence variants present in both plant and animal genomes (Kwok, 2001). They are present across the genome, within coding, non-coding and intergenic regions of the genome (Jiang, 2013). They gained tremendous importance as a third-generation molecular marker for a wide range of biological applications of marker-assisted and genomic selection, associations and QTL mapping, haplotype and pedigree analysis and cultivar identification (Lee et al., 2008; McCouch et al., 2010; Subbaiyan et al., 2012).

To this end, our study will appear as a first and new approach for the discovery of selective SNP loci of CA. Prior studies have revealed that CA is a complex quantitative trait (Liu C. et al., 2015). Therefore, the identification of marker loci within the parental genomes of japonica hybrid rice can ultimately be utilized to understand the biological and genetic factors of CA. Here, we aim to develop a reliable strategy to select and determine the superior parents of hybrid rice based on the presence of CA loci, which can be achieved by using SNP data with combinations of SNP and phenotype data. The main objectives of this study were as follows: (1) to dissect japonica parents for genome-wide SNPs; (2) to evaluate japonica parents for GCA effect; and (3) to associate the identified SNPs of CMS and restorer lines with the CA of parents to determine genome-wide favorable or unfavorable SNP loci of CA related to yield-related traits.

## Materials and methods

### Library construction and sequencing

The total DNA of 18 japonica rice was rice extracted from fresh leaves (10–20 mg) selected at the seedling stage using the Qiagen DNeasy Plant Mini Kit. The experimental procedure for DNA isolation was the same as that previously described (Stein et al., 2001). Following the recommended protocol of GBS, optimized by Poland et al., a restriction digest buffer NEB Buffer 4 and a core set of restriction enzymes, *Pst*I (CTGCAG) and *Msp*I (CCGG), were added to each sample to obtain a uniform distribution of cut sites across the rice genome (Poland J. A. et al., 2012). The DNA library fragment size and library concentration were analyzed using a bioanalyzer (Agilent) machine (Parson et al., 2013). To inactivate the enzymes, the samples were incubated twice at 37°C for 2 h and at 65°C for 20 min. A set of already developed adapters with 500 ng of high-quality DNA was attached to the sample ends preceding the ligation reaction. The ligated samples were pooled and selected on a 1.5% agarose gel (200–300 bp) followed by PCR-amplification in a single tube. The resulting fragments in the DNA library were sequenced using the Ion Torrent (PGM) sequencing machine with Ion Torrent kits (PGM 200 Kit v2) following the manufacturer specifications (Life Technologies, Carlsbad, CA, and U.S.A.). The sequencing data obtained in our experiment have been deposited into the NCBI short read archive (SRA) under the study accession number:SRR2758809.

### Short read mapping, SNP discovery and annotation

The FASTQ format sequence reads obtained from the sequencing machine were treated for quality control. For this, the adopter sequences and low quality reads were analyzed and trimmed. The cleaned sequences of all 18 samples were aligned onto the Nipponbare genome (IRGSP-1.0) (http://rapdb.dna.affrc.go.jp/) with bowtie 2 software (Langmead, 2010). The –M4 –very-sensitive local parameter for mapping was adopted (-D 20 -R 3 -N 0 -L 20 -i S,1,0.50), where if the alignment is greater than 4, it will output only 1 alignment at a random level. The reads that mapped successfully once onto a reference genome were considered to be uniquely mapped reads, whereas the reads mapped onto multiple locations were considered to be multiple-mapped reads. The short reads that could not be mapped onto any part of the reference were called unmapped reads. The GBS data were further used for SNP discovery using the Tassel-GBS application with default parameters (Glaubitz et al., 2014). The SNPs that evolved repeatedly were deleted from the final results.

The physical locations of the discovered SNPs were annotated by SNPEff software (Cingolani et al., 2012). The SNPs that were situated within exon, intron and other corresponding regions were classified in detail. We also grouped the identified SNPs into different classes of polymorphisms, such as transition/transversions mutations and gene-base synonymous and non-synonymous nucleotide base mutations.

### Field experiment and GCA calculation

In our experiment, all the plant materials belonged to Japonica rice, comprising nine CMS and nine restorer lines. These parental lines are widely used in China for the commercial breeding of three-line hybrid rice. For hybrid development, all the CMS and restorer lines were planted and manually crossed in a set of 9 × 9 combinations following the NCII mating design (North Carolina mating design II) (Shukla and Pandey, 2008). At the end of rice-cropping season, a population of 81 hybrids was obtained. The resultant hybrid seeds were grown and evaluated the next year at the Jiangpu research station, Nanjing Agricultural University, Nanjing China, with randomized complete block designs in four replications. The seeds of hybrid and their parents (restorer lines, and maintainer lines instead of CMS lines) were sown in the rice nursery in May 10, 2015. After 30 days, the seedlings of each hybrid combination were transplanted to paddy fields. Each plot in the paddy contained 4 rows with 8 plants per row. Twenty centimeter row-to-row and plant-to-plant spacing for all hybrids and their parents was maintained. Recommended field management and cultural practices were applied accordingly to the local environment. At maturity, six plants from each plot were randomly selected to calculate the phenotypic data of 12 yield-related traits. The phenotypic data of the traits were tested for significance using the statistical method of analysis of variance, along with the coefficient of variation (CV %) as described by Gomez and Gomez (1984). The GCA effect values of each CMS and restorer line for the traits were calculated by using the phenotypic data of the developed hybrids, as per the Griffing model (1956), following method-2 (Mo, 1982). The significance of the parental GCA value was tested with an LSD test at a *P* < 0.01.

### Association analysis

To identify the SNP locus/loci associated with the CA of the yield-related traits, an association analysis between the identified SNPs and CA of 12 parental traits was performed using a computational software called CA screen 1.0 operated in the MATLAB language and developed by our laboratory^*^ (Liang et al., 2010). The script of our association method follows the principle of single-marker analysis (SMA). This method of association led to the statistically significant identification of SNPs and their effect on CA in homozygous and heterozygous associations. Moreover, we explain the principle of this association model corresponding to the research article provided, where we developed 81 F_1_ combinations by crossing nine CMS with nine restorer lines. Now, at a given locus of SNP, if the parental lines of 41 F_1_ hybrids possess the heterozygous SNP genotype (for example A-G, A-T), and the parental lines of the remaining 40 F_1_ hybrids possess the homozygous SNP genotype (A-A or G-G). Now, after association, if the average trait value of the 41 heterozygous crosses is significantly greater or less than the average trait value of the 40 homozygous crosses, then the SNP marker associated with the CA of the trait is significantly positive or negative. If the difference in the trait value in the heterozygous association is positive and we observed a positive effect on the CA of the trait, we consider this SNP locus to be a favorable associated marker genotype of the elite CA for the trait, and *vice versa*.

In our study, to avoid spurious associations, the significance of the associations was assessed by using a *t*-test at *P* < 0.01 (Pradeep et al., 2007). Furthermore, coefficient of determination (*R*^2^) was calculated to determine the percentage of phenotypic variation explained by each SNP marker.

### Selection of functional SNPs

To determine the substantial number of functional SNPs, we selected associated SNPs based on their genomic positions. Only the SNP loci situated within the coding region of the genome (synonymous, non- synonymous and splice variants) were considered. Furthermore, in the NCBI, the database GENE was used to search the corresponding genes with associated SNPs for their nomenclature, chromosomal location, gene product, protein domain, and expected functions.

## Results

### Short read mapping, SNP discovery, and distribution

**Figure 1:**
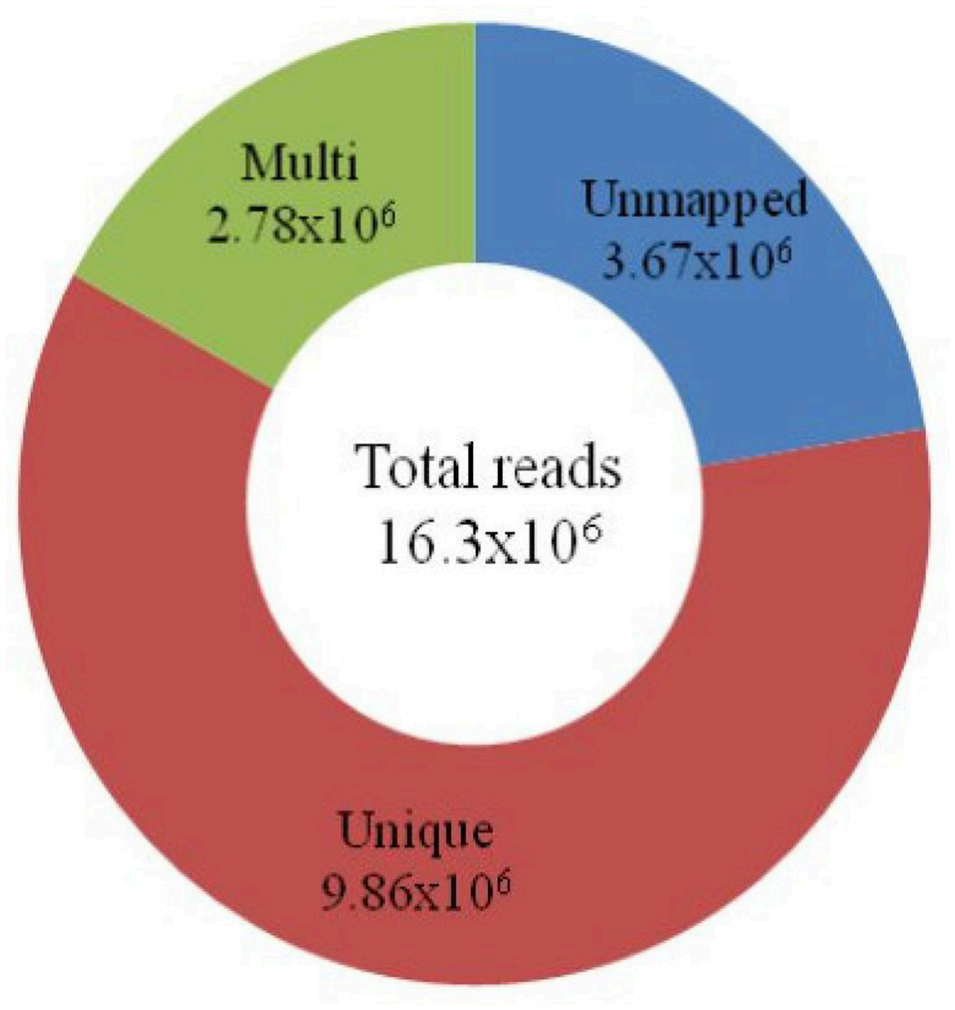
**Classification of total short reads mapped onto the Nipponbare genome**. The total number of reads (16.3 × 10^6^) obtained through the GBS of 18 japonica rice is in the center of the circle. The number of unmapped reads (3.67 × 10^6^) is shown in *blue*. The 9.86 × 10^6^ short reads, in *brown*, are mapped uniquely onto the reference genome. The *green* circle represents the multiple (2.78 × 10^6^) mapping of reads on chromosomes.

Using a GBS assay, approximately 16 million (16.3 × 10^6^) short reads were constituted, which comprised 4.7 GB of data. After removal of low quality reads, the sequenced data were mapped onto the Nipponbare reference genome. The mapping result indicated that 60.4% (9.86 × 10^6^) of the short reads were uniquely mapped onto the 12 chromosomes of the Nipponbare genome. Approximately 17% (2.78 × 10^6^) of the short reads were mapped more than once (Figure 1). The remaining 22.4% (3.67 × 10^6^) of the short reads could not be mapped onto any part of the reference genome.

**Figure 2:**
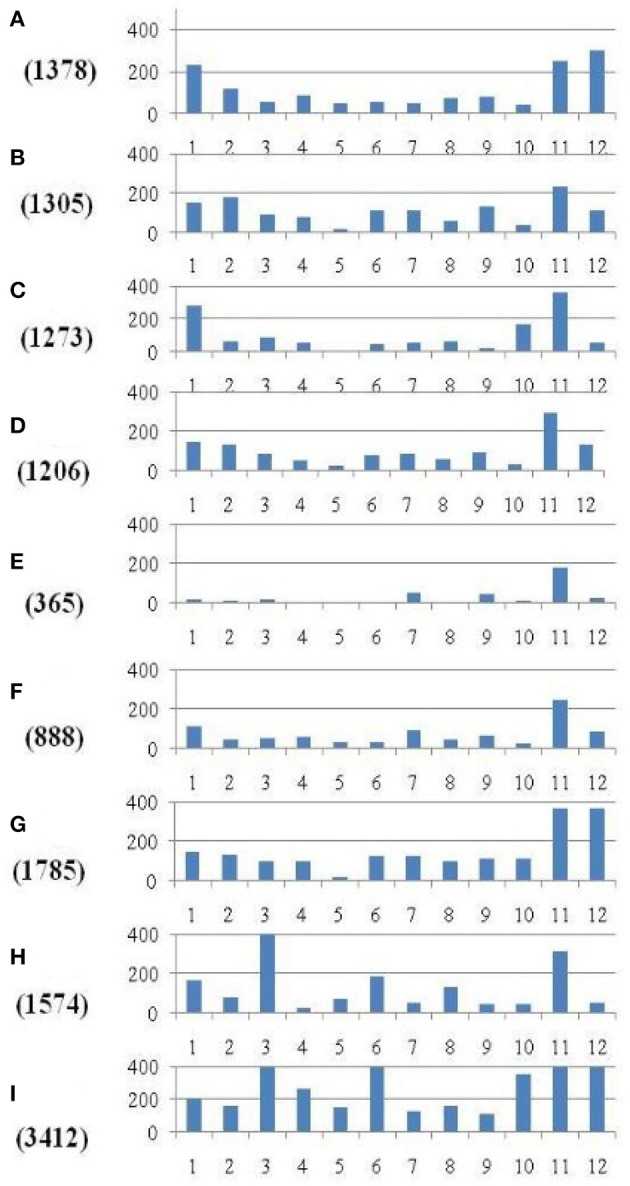
**Distribution of SNPs identified between each of the nine CMS lines and the Nipponbare reference genome**. The *x*-axis represents the chromosome numbers, while the *y*-axis represents the total number of SNPs. The nine CMS lines are **(A)** 95122A, **(B)** 90167A, **(C)** 863A, **(D)** A171, **(E)** Aizhixiang A, **(F)** 18A, **(G)** Zhe 04A, **(H)** Chunjiang 19A, and **(I)** Chunjiang 18A. The figure along the*y*-axis in the parentheses is the total number of identified SNPs in the nine CMS lines.

**Figure 3:**
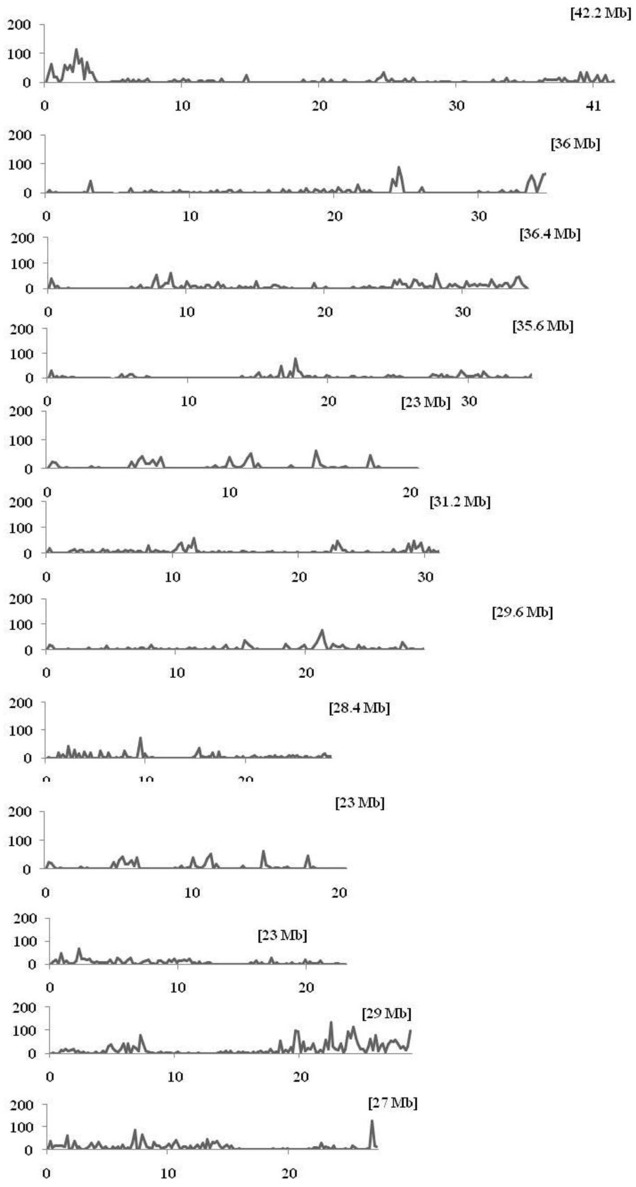
**Density distributions of SNPs detected among the nine CMS lines and Nipponbare in the 12 rice chromosomes**. The x-axis indicates the physical distance of each chromosome, split into 200-kb windows. The total size of the individual chromosome is shown in the bracket. The y-axis indicates the total number of SNPs.

Within the mapped sequenced reads of the nine CMS and nine restorer lines, a total of 39,001 SNPs were discovered at 11,085 sites, including 13,186 and 25,815 in the CMS and restorer lines, respectively. Despite having the same coverage in the genomes, the SNPs identified in the restorer lines were double those in the CMS lines. Of the nine CMS lines, 13,186 SNPs were identified, where the number of SNPs within the CMS lines ranged from 365 in Aizhixiang A to 3412 in Chunjiang 18A (Supplementary Table 1). The discovered SNPs in the CMS lines were distributed non-randomly on the 12 chromosomes, where chromosome 11 occupied a maximum (2740) and chromosome 5 occupied a minimum (381) of SNP numbers (Figure 2). The density distributions of the discovered SNPs in the CMS and restorer lines were explored by calculating the SNP frequency within a 200 kb genomic region. The average SNP densities between the CMS lines per 200 kb window were 7.5. Likewise, the density distribution varied across the chromosomes, where chromosome 9 possessed the highest SNP densities of 18.3, while chromosome 5 possessed the lowest SNP densities of 3.2. This phenomenon was also found to be uneven within the individual chromosome. For example, chromosome 11 in the CMS lines was found to be dense (750 SNPs) from the 25 to 28.8 MB genomic region, but scant SNPs (48 SNPs) were observed from the 11 to 16 MB genomic region. Similarly, on chromosome 6, the 10 MB region from 1.8 to 11.8 contained 542 SNPs, but on the same chromosome, the region from 16.6 to 22.4 MB had only 53 SNPs. On chromosome 9, we observed 34 high-density SNP sites with >30 SNPs per 200 kb region (Figure 3).

**Figure 4:**
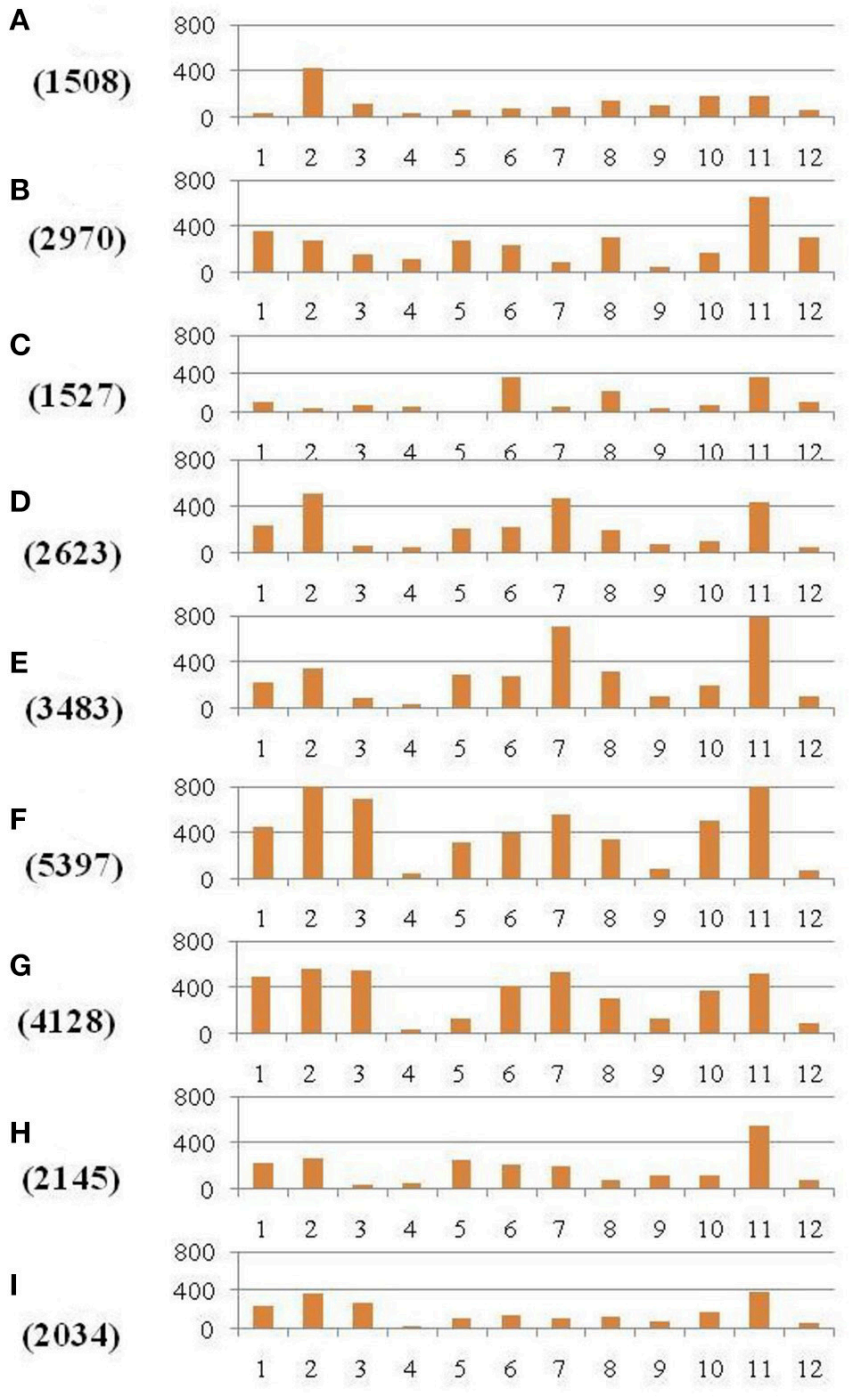
**Distribution of SNPs identified between each of the nine restorer lines and the Nipponbare reference genome**. The *x*-axis represents the chromosome numbers, whereas the *y*-axis represents the total number of SNPs. The nine restorer lines **(A)** R4179, **(B)** LC64, **(C)** LC109, **(D)** Yanhui R50, **(E)** Yanhui R8, **(F)** LR5, **(G)** LR27, **(H)** Shenhui 254, and **(I)** C4115. The figure along the *y*-axis in parentheses is the total number of identified SNPs in the nine restorer lines.

**Figure 5:**
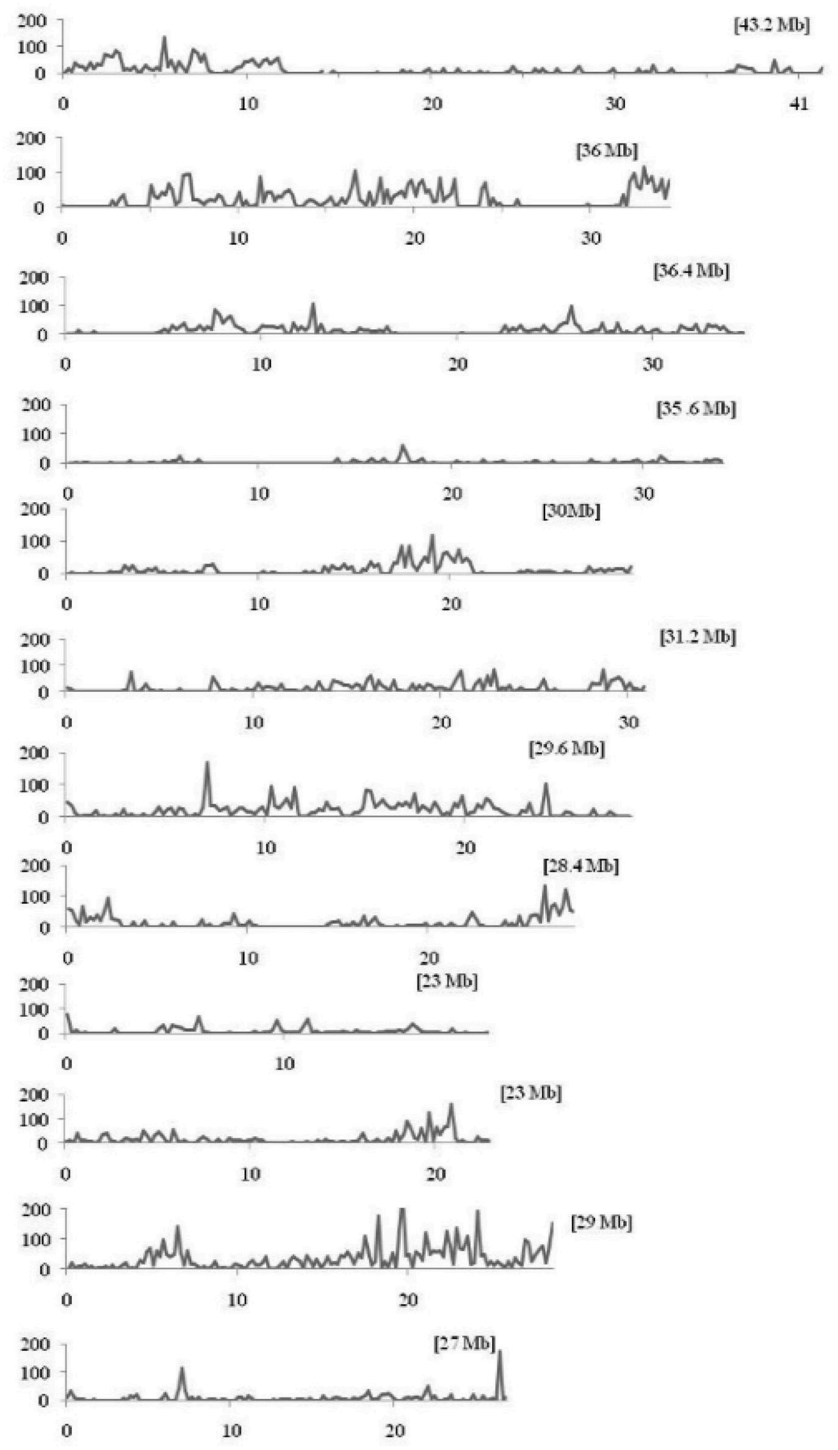
**Density distributions of SNPs detected among the nine restorer lines and Nipponbare in the 12 rice chromosomes**. The x-axis indicates the physical distance of each chromosome, split into 200-kb windows. The total size of individual chromosomes is shown in the bracket. The y-axis indicates the total number of SNPs.

The discovered SNPs in nine restorer lines were counted as 25,815. These SNPs ranged from 1508 in R4179 to 5397 in LR5 (Supplementary Table 2). Due to the different chromosomal lengths, the numbers of SNPs across individual chromosomes varied. For example, for all the restorer lines, chromosome 11 was found to be rich in SNP number with 4845 SNPs, whereas a low SNP number (464) was found on chromosome 4 (Figure 4). The average densities of the SNPs among the nine restorer lines were 15 SNPs per 200 kb genomic region. Such densities across chromosomes were non-randomly distributed. The highest (35.6) SNP density was observed on chromosome 11, whereas the lowest (2.7) was observed on chromosome 4. Such densities were also found to be uneven within the individual chromosomes. In chromosome 2, the region between 5 and 22.2 MB had an unusually high (2796 SNPs) SNP density, whereas the region between 26 and 32.4 MB had a very low (24 SNPs) SNP density. The same results were observed on chromosome 11, where a high (3508 SNPs) and long SNP interval was detected between the 16 and 28.4 MB genomic region (Figure 5). A small (120 SNPs) and short interval was also found from the 0.4 to 4 MB region of the same chromosome. On chromosome 11 of the restorer lines, we identified a maximum of 57 high-density SNP sites, with >30 SNPs per 200 kb region.

### Annotation of SNPs

**Figure 6:**
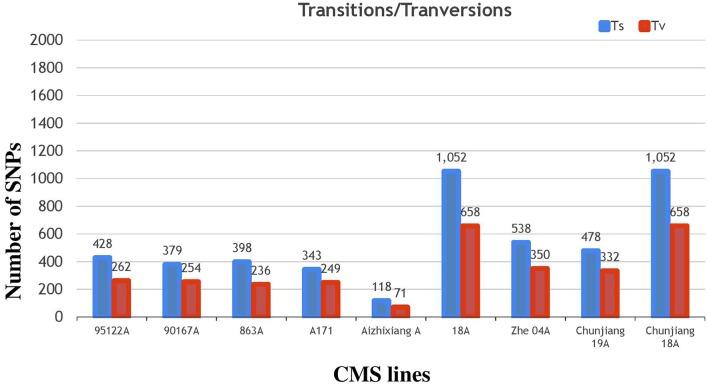
**Classifications of nucleotide base substitutions in the SNPs detected in the nine CMS lines**.

The annotation of the Nipponbare rice was used to locate the distribution pattern of the identified SNPs within various genomic regions. Overall, a similar SNP distribution was observed in the CMS and restorer lines. Of the 13,186 SNPs discovered in the nine CMS lines, 7582 (57.5%) were found within intergenic spaces. The other 3212 (24.3%) and 2649 (20%) were in the intron and exon regions, respectively. Among the total SNPs in the coding regions, synonymous were counted as 1155 (8.7%), while non-synonymous were further divided into missense, 1214 (9.2%), and nonsense, 27 (0.2%) (Table 1, Supplementary Table 3). Moreover, the detected SNPs of the nine CMS lines were also categorized as transition (C/T and G/A) and transversion (G/T, T/A, A/C, C/G) nucleotide bases (Supplementary Table 5). In our results, the average ratio between the transitions and transversions was 1.4. The number of transition substitutions was significantly higher than that of transversion substitutions in the identified SNPs of all nine CMS lines (Figure 6).

**Table 1:** **Annotation of single nucleotide polymorphisms (SNPs) between nine CMS lines and the Nipponbare reference genome**.

**CMS lines**	**Total**	**Intergenic**	**Splice site**	**Intron**	**Exon**	**Synonymous**	**Non-synonymous**	
							**Missense**	**Nonsense**
95122A	1,378	793	13	357	276	119	139	–
90167A	1,305	770	19	328	265	121	110	2
863A	1,273	700	18	271	308	130	132	3
A171	1,206	702	12	277	259	120	116	3
Aizhixiang A	365	219	1	84	67	30	29	1
18A	888	516	27	213	169	80	73	2
Zhe 04A	1,785	1,026	20	399	362	167	158	6
Chunjiang 19A	1,574	884	21	430	288	118	149	1
Chunjiang 18A	3,412	1972	49	853	655	270	308	8

**Figure 7:**
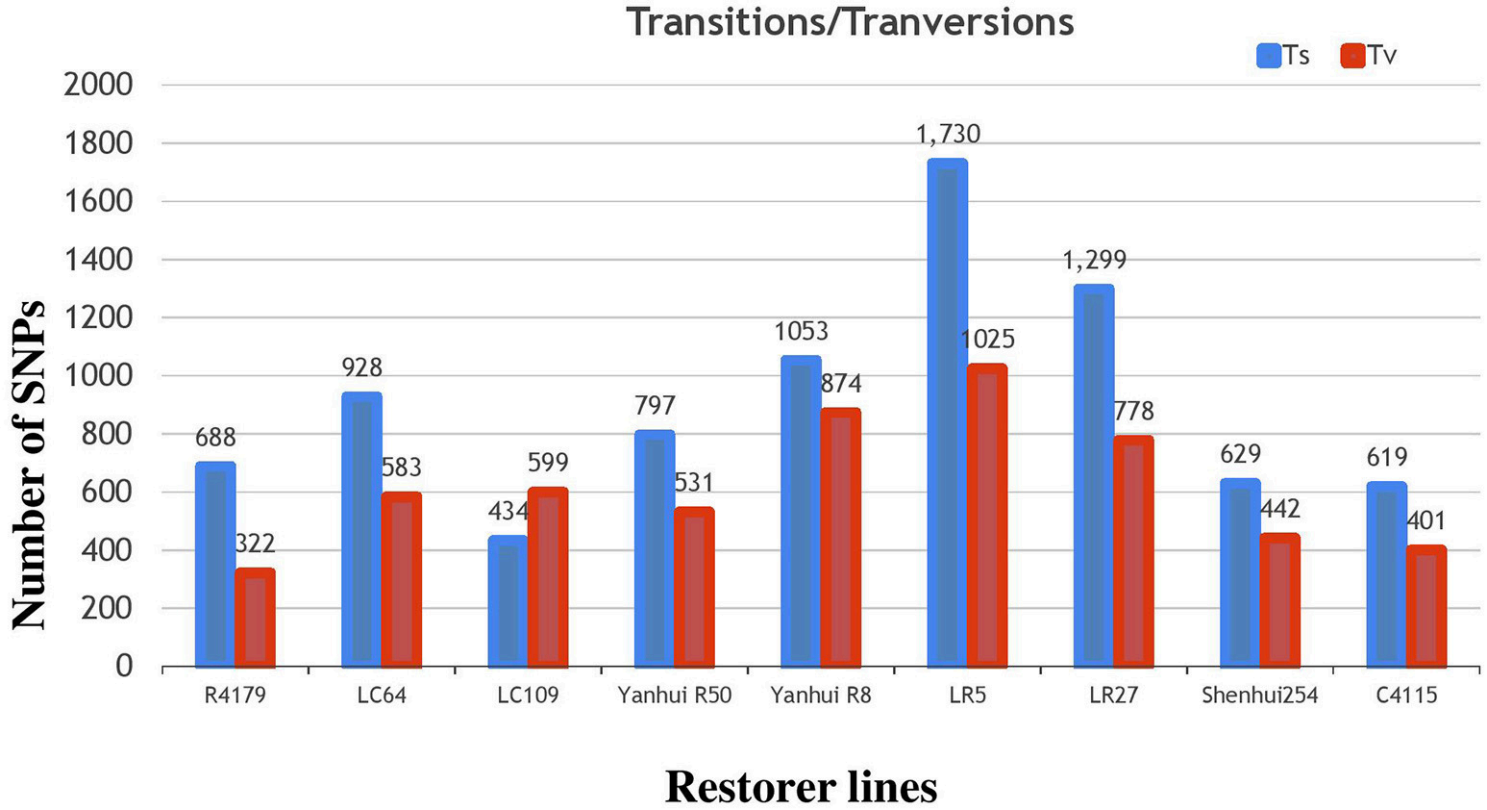
**Classifications of nucleotide base substitutions in the SNPs detected in the nine restorer lines**.

Subsequently, the identified SNPs of the restorer lines were also annotated, where 14,970 (58%) were situated in the intergenic region of genome. However, 6226 (24%) and 5377 (20%) were detected within the intron and exon regions (Table 2, Supplementary Table 4). Of the SNPs in the coding regions, 2189 (8.4%) led to synonymous while 2711 (10.5%) led to non-synonymous amino acid changes with a protein-altering effect on genes. The average ratio of the transitions and transversions in the identified SNPs was also 1.4 (Supplementary Table 6). The similar result of higher transition substitutions was also observed in the identified SNPs of all nine restorer lines (Figure 7).

**Table 2:** **Annotation of single nucleotide polymorphisms (SNPs) between nine restorer lines and the Nipponbare reference genome**.

**Restorer lines**	**Total**	**Intergenic**	**Splice site**	**Intron**	**Exon**	**Synonymous**	**Non-synonymous**	
							**Missense**	**Nonsense**
R4179	1,508	850	16	342	355	142	174	1
LC64	2,970	1,774	40	639	622	276	303	3
LC109	1,527	904	14	338	300	130	137	1
Yanhui R50	2,623	1,516	27	604	538	205	369	3
Yanhui R8	3,483	2,057	37	856	696	291	334	4
LR5	5,397	3,080	63	1,372	1,118	441	529	4
LR27	4,128	2,364	44	1,058	828	318	420	1
Shenhui 254	2,145	1,288	21	479	454	195	205	4
C4115	2,034	1,137	20	538	466	191	218	1

### Performance of the GCA of parents for 12 yield-related traits

The GCA performance of the 18 parents of hybrid rice for 12 yield-related traits is summarized in Table 3. Among the nine CMS lines, genotype Zhe04 exhibited the highest GCA effect value for plant height, whereas A171 had the highest GCA effect for days to heading and seed width. In terms of the panicle length, the number of panicles per plants and the number of grains per panicle, CMS line 863A was a good general combiner due to the highly significant and positive GCA effect value. CMS 18A recorded the maximum GCA effect value for seed thickness and grain yield per plot, whereas 90167A revealed the highest GCA effect value in terms of seed length and 1000-seed weight. Parent AizhixiangA and Chunjiang18A had the maximum GCA effect in terms of number of panicles per plant and seed setting rate, respectively (Table 3).

**Table 3:** **GCA effect values of 18 parental lines for 12 yield-related traits**.

**Parental lines**	**PH**	**GW**	**GL**	**GT**	**1000-GW**	**GYPP**	**DH**	**PL**	**NSPP**	**NPPP**	**NFGPP**	**SSR**
**CMS LINES**												
95122A	3.94a	−0.12b	0.13a	−0.07c	−0.79cd	−0.23b	3.64b	−0.56cd	−1.77bc	−0.59b	5.85a	−0.24bc
90167A	−1.49bc	0.12a	0.18a	0.05a	1.29a	0.21ab	−4.07d	0.57c	16.65a	−0.58b	14.07a	−0.26c
863A	3.95a	0.01a	−0.01a	0.00b	−0.25c	−0.57b	3.12bc	4.14a	23.93a	−0.86b	16.07a	−0.26c
A171	−11.98d	0.19a	−0.03a	0.05ab	0.84ab	−0.48b	7.84a	−3.99e	−43.27c	0.38ab	−23.45b	−0.19b
Aizhixiang A	−0.23b	−0.13b	−0.16b	−0.01b	−0.68c	0.46a	−6.10e	2.50b	−40.83c	1.84a	−22.46b	−0.22b
18A	0.81b	0.05a	−0.04ab	0.08a	0.15bc	0.80a	1.37c	−1.70de	6.68a	0.89a	7.09a	−0.25c
Zhe 04A	4.77a	0.02a	−0.21b	−0.03bc	0.15b	−0.10b	7.23ab	−1.01d	19.57a	−1.15b	3.95a	−0.30c
Chunjiang 19A	2.61ab	−0.05ab	0.13a	−0.05c	−1.30d	−0.03b	−7.76e	0.15c	18.06a	−0.04b	4.31a	−0.30c
Chunjiang 18A	−2.386cd	−0.10b	0.02a	−0.01b	0.58b	−0.04b	−5.269de	−0.10c	0.95ab	0.10b	−5.45ab	2.04a
**RESTORER LINES**												
R4179	−3.76c	−0.20b	−0.05b	−0.02c	−0.69de	0.11b	−1.63d	−2.57c	−12.92c	1.52a	−16.05b	0.01b
LC64	−4.46cd	−0.06ab	−0.47c	−0.06c	−1.71e	0.74ab	−6.93e	0.84b	56.66a	0.41a	19.24a	−0.14d
LC109	−8.21d	0.034a	−0.38c	0.06ab	0.30bc	−0.78c	−6.88e	−1.43c	−69.99d	−0.18b	−67.17c	−0.10d
Yanhui R50	−0.28b	0.11a	0.34a	0.02b	0.03c	−0.73c	4.56b	0.66b	11.30b	−1.75b	14.71ab	0.01bc
Yanhui R8	−0.93bc	0.19a	0.51a	0.06a	2.08a	−0.00b	4.00bc	0.28bc	−10.18c	−0.79b	−1.34b	0.05a
LR5	5.22a	−0.03a	−0.04b	−0.04c	−0.00c	1.03a	0.53cd	0.55b	21.54b	1.35a	33.09a	0.05ab
LR27	6.06a	−0.10b	0.09b	−0.06c	−0.32c	0.00b	1.23c	1.44ab	20.83b	−0.05ab	20.64a	0.02b
Shenhui254	4.98ab	0.06a	−0.27bc	0.08a	0.72b	−0.11b	8.39a	−2.12c	−18.31cd	1.20a	−24.52bc	−0.03cd
C4115	1.39b	−0.00a	0.27ab	−0.04c	−0.40cd	−0.23bc	−3.29de	2.34a	1.08bc	−1.71b	21.41a	0.11a

*The CMS and restorer line values followed by different letters are significantly different at P < 0.01. PH, plant height; GW, grain width; GL, grain length; GT, grain thickness; 1000-GW, 1000-grain weight; DH, days to heading; PL, panicle length; NPPP, number of panicles per plant; NSPP, number of spikelets per panicle; NFGPP, number of filled grains per panicle; SSR, seed setting rate; GYPP, grain yield per plot*.

The GCA assessment of the restorer lines indicated that LR27 recorded the maximum GCA effect value for plant height, whereas Shenhui254 recorded the maximum GCA effect value for days to heading. The restorer C4115 possessed higher GCA effect values for panicle length, seed thickness and seed-setting rate. In terms of seed length, seed width and 1000-seed weight, restorer Yanhui R8 recorded the highest GCA effect value. The parent LC64 exhibited a greater value for the number of spikelets per panicle, whereas LR5 exhibited a greater GCA effect for the number of filled grains per panicle and grain yield per plot. The restorer R4179 revealed the maximum GCA for the number of panicles per plant (Table 3).

### Genome wide association analysis for the identification SNP loci associated with CA

**Figure 8:**
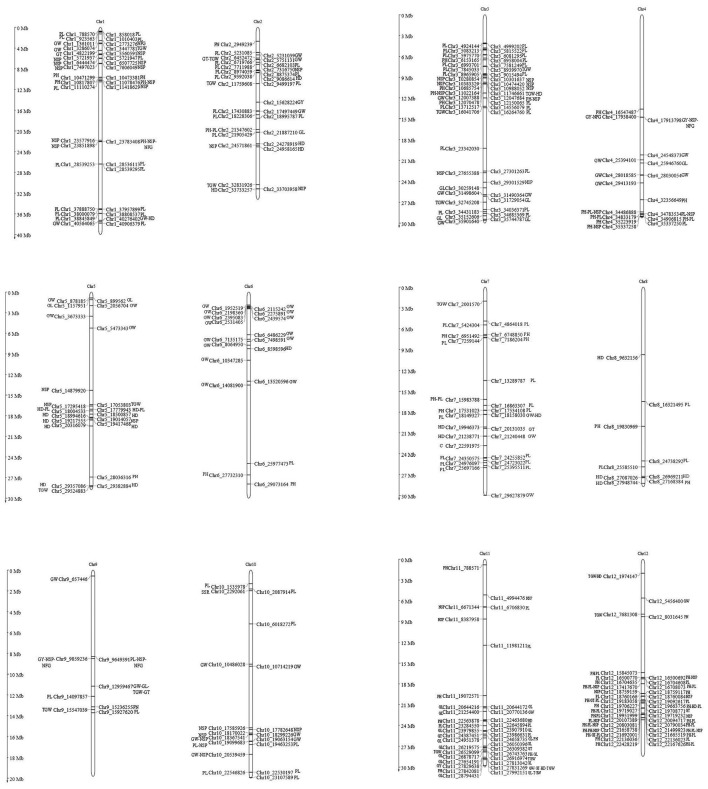
**Graphical representations of all the associated 362 SNP loci of CA and their corresponding chromosomal positions**. SNP positions associated with the CA of traits are shown by alphabet, PH, plant height; GW, grain width; GL, grain length; GT, grain thickness; TGW, 1000 grain weight; GY, grain yield; HD, heading days; PL, panicle length; PN, panicle number per plant; NSP, number of spikelets per panicle; NFG, number of filled grains per panicle; SSR, seed setting rate.

The main objective of our study was to identify SNP locus/loci of CA *via* association analysis; the discovered 39,001 SNPs at 11,085 genomic positions were integrated with the parental CA of 12 yield-related traits at *P* < 0.01. We revealed a total of 362 SNP locus/loci with the CA of parental traits that caused a positive or negative effect on F_1_ trait performances. The overview and detailed information of the identified SNP loci of CA for the following traits are presented in Figure 8 and Supplementary Table 7.

#### Plant height

Fifty-three SNP loci situated on 9 different chromosomes (Chr1 Chr2, Chr3, Chr4, Chr6, Chr7, Chr8, Chr11, and Chr12) displayed significant associations with the CA of plant height (Table 4, Supplementary Table 7). Of these associated loci, 18 exhibited a positive and 35 exhibited a negative effect on the CA of plant height. The positively associated loci increased the CA by 6.6%, whereas the negatively associated loci reduced the CA by 11%. Among all the parents, the restorer LC64 possessed a maximum of 35 negative CA loci of plant height in its sequence genome (Table 5). On an individual basis, SNP loci on chromosomes 11 and 7 with A/G and T/A alleles caused the most prominent positive effect on the CA of the trait, 8.8 and 7.9%, respectively.

**Table 4:** **Number of SNP loci significantly associated with CA and percentage of increase/decrease in the trait values**.

**Traits**	**Number of associated SNP loci**	**Heterozygous group**		**Homozygous group**		**Average percent heterosis at the heterozygous locus group^a^**
		**Number of total F_1_ crosses**	**Average trait value of F_1_ crosses**	**Number of total F_1_ crosses**	**Average trait value of F_1_ crosses**	
PH	53	871	107.4	3,422	110	−4.9
GW	44	593	2.2	2,971	2.1	4.9
GL	27	544	8.3	1,643	8.2	−0.2
GT	10	189	3.7	621	3.5	6.1
1000 GW	19	413	26.2	1121	25.5	2.8
GYPP	6	84	25.5	321	31	−16.7
DH	27	808	110.6	1,379	105.4	10.2
PL	110	1,849	23.6	7,061	22.4	5.9
NPPP	5	68	11.4	337	9.6	19.2
NSPP	53	914	229	3,379	281.9	−17.7
NFGPP	7	114	177.4	453	222	−25.2
SSR	1	10	0.65	71	0.8	−19.1

a *Heterosis at heterozygous locus group (%) = (average of trait value in F1 heterozygous locus group—average of trait value in F1 homozygous group)/average of trait value in F1 homozygous group × 100. PH, plant height; GW, grain width; GL, grain length; GT, grain thickness; 1000-GW, 1000-grain weight; GYPP, grain yield per plot; DH, days to heading; PL, panicle length; NPPP, number of panicles per plant; NSPP, number of spikelets per panicle; NFGPP, number of filled grains per panicle; SSR, seed setting rate*.

**Table 5:** **Number of associated SNPs of CA in the parental genomes for 12 yield-related traits**.

**CMS lines**	**PH**	**GW**	**GL**	**GT**	**1000-GW**	**GYPP**	**DH**	**PL**	**NSPP**	**NPPP**	**NFGPP**	**SSR**
95122A	3	2	1	1	4	2	2	1	2	2	2	–
90167A	–	2	3	–	2	2	1	2	4	1	4	–
863A	1	4	11	2	3	3	5	5	2	1	3	–
A171	–	3	13	3	9	3	4	2	1	1	2	–
Aizhixiang A	2	–	2	–	–	1	–	3	6	–	3	–
18A	2	1	1	–	3	3	2	4	5	2	5	–
Zhe 04A	2	1	7	1	3	2	4	3	2	–	1	1
Chunjiang 19A	3	–	10	–	3	–	4	7	2	–	1	–
Chunjiang 18A	4	34	12	1	3	3	5	24	2	–	2	1
Restorer lines												
R4179	1	8	8	2	4	4	3	13	4	–	2	–
LC64	35	5	4	5	3	4	15	35	47	–	4	–
LC109	6	2	7	–	5	3	1	6	2	–	2	1
Yanhui R50	15	7	5	2	4	4	14	11	7	–	3	–
Yanhui R8	13	9	3	1	3	3	14	16	7	–	3	–
LR5	17	8	4	2	7	4	14	62	33	2	3	–
LR27	13	6	6	4	5	2	14	78	14	–	3	–
Shenhui254	4	7	5	–	4	–	4	6	3	1	3	–
C4115	8	1	–	1	5	–	7	10	2	–	1	–

*Symbol (–) indicates that none of the associated SNP loci of CA are present in the genome of parents. PH, plant height; GW, grain width; GL, grain length; GT, grain thickness; 1000-GW, 1000-grain weight; GYPP, grain yield per plot; DH, days to heading; PL, panicle length; NPPP, number of panicles per plant; NSPP, number of spikelets per panicle; NFGPP, number of filled grains per panicle; SSR, seed setting rate*.

#### Grain width

Forty-four SNP loci were distributed over 11 different chromosomes and showed significant associations with the CA of grain width (Table 4, Supplementary Table 7). Of them, 40 showed an increment (5.8%), whereas 4 exhibited a reduction (3.5%). The CMS Chunjiang 18A had a maximum of 34 favorable CA loci in the genome for the CA of grain width (Table 5). On an individual basis, SNP loci on chromosomes 6 and 5 with A/T and T/C alleles caused the maximum positive effect on the CA of the trait, 11 and 7.6%, respectively.

#### Grain length

Twenty-seven SNP loci distributed across 10 different chromosomes revealed significant associations with the parental CA of grain length (Table 4, Supplementary Table 7). Among the associated loci, 17 conferred a positive effect of 5%, whereas 10 conferred a negative effect of 8%. For the number of associated SNP loci within the parents, the CMS A171 had a maximum of 13 favorable CA loci for grain length (Table 5). On an individual basis, SNP loci on chromosomes 7 and 11 with C/T and G/A alleles caused the maximum positive effect on the CA of the trait, 6 and 5.9%, respectively.

#### Grain thickness

Ten SNP loci on 8 different chromosomes (Chr2, Chr3, Chr4, Chr5, Chr6, Chr7, Chr10, and Chr11) revealed significant relationships with the CA of grain thickness (Table 4, Supplementary Table 7). The effect of these loci on the CA explained that, 9 loci caused a 7.2% increment, whereas one locus caused a 4.9% reduction in the CA of grain thickness. Among the sequenced genome of the parents, the restorer LC64 exhibited a maximum of 5 positive CA loci (Table 5). On an individual basis, SNP loci on chromosomes 7 and 12 with C/G and A/G alleles caused the maximum positive effect on the CA, increasing its value by 16.9 and 7.4%, respectively.

#### 1000-grain weight

Nineteen SNP loci situated over 9 different chromosomes (Chr1, Chr2, Chr3, Chr5, Chr6, Chr7, Chr9, Chr11, and Chr12) recorded significant associations with the CA of 1000-grain weight. Of them, 14 explained positive (5.8%), whereas 5 explained negative (5.4%), associations with the CA of the trait (Table 4, Supplementary Table 7). The parental genome of CMS A171 contained a maximum of 9 positive CA loci of 1000-grain weight (Table 5). On an individual basis, SNP loci on chromosomes 5 and 2 caused the maximum positive effect, increasing the CA trait by 7.8 and 5.5%, respectively.

#### Grain yield

Five SNP loci detected on 4 different chromosomes (Chr2, Chr3, Chr9, and Chr11) were found to be significantly associated with the CA of grain yield (Table 4, Supplementary Table 7). Among them, one locus had a positive effect of 26.3%, whereas 4 loci had a negative effect of 27.5%. The restorer line LC64 contained a higher number of negative CA loci for grain yield (Table 5). Among all the associated SNPs, an SNP locus on chromosome 2 with a G/A allele had a favorable (26.3%) effect on the CA value.

#### Days to heading

Twenty-seven SNP loci situated on 9 different chromosomes (Chr1, Chr2, Chr3, Chr5, Chr6, Chr7, Chr8, Chr11, and Chr12) revealed significant associations with the CA of days to heading (Table 4, Supplementary Table 7). Among them, 22 had a positive effect of 7.8% on the CA, whereas 5 caused a negative effect of 7.6% on the CA of the trait. We found that the restorer LC64 had a maximum number of positive CA loci in days to heading (Table 5). On an individual basis, SNP loci on chromosomes 5 and 1 with A/G and G/C alleles contributed the maximum effect to the CA in terms of days to heading, 10.2 and 9.7%, respectively.

#### Panicle length

One hundred and ten SNP loci distributed over all 12 chromosomes revealed significant associations with the CA of panicle length. Among them, 77 exhibited a positive (16.2%) contribution, whereas 33 exhibited a negative reduction in the CA (Table 4, Supplementary Table 7). For all the sequenced parents, the restorer LR27 had a maximum number of 71 favorable and 7 unfavorable CA loci in the panicle length (Table 5). On an individual basis, SNP loci on chromosomes 9 and 3 with A/C and T/C alleles showed the maximum positive effect of 34 and 25%, respectively.

#### Panicle number per plant

Five SNP loci located on 4 different chromosomes (Chr2, Chr5, Chr9, and Chr11) showed significant associations with the CA of panicle number per plant (Table 4, Supplementary Table 7). Of them, 4 had a positive (29.3%), while one locus had a negative (21.9%), effect on the CA of the trait. CMS 18A contained a maximum of 4 negative loci, causing a reduction in the CA value of the panicle number per plant (Table 5). Among all the SNP loci, the variants on chromosomes 5 and 9 having G/A and A/T alleles caused the maximum improvement in the CA of 39 and 31%, respectively.

#### Number of spikelet's per panicle

Fifty-three SNP loci distributed over 10 different chromosomes were found to be significantly associated with the CA of number of spikelet's per panicle (Table 4, Supplementary Table 7). Of these, 2 loci had a positive (3.7%) and 51 had a negative (4.2%) effect on the CA of the trait. The restorer LC64 had a maximum of 47 negative CA loci (Table 5). Two associated loci on chromosomes 11 and 2 with A/G and C/T alleles exhibited the maximum positive effect on the CA of 19 and 15%, respectively.

#### Number of filled grains per panicle

Seven SNP loci were found to be associated with the CA of number of filled grains per panicle. These loci were distributed on 4 different chromosomes (Chr1, Chr2, Chr4, and Chr9) (Table 4, Supplementary Table 7). Of these, one locus contributed positively (20%), whereas the remaining 6 loci negatively affected (26%) the CA of the trait. It is noteworthy that, CMS 18A had a higher (5) number of negative CA loci in its genome of CA (Table 5). An SNP locus on chromosome 2 with a C/T allele caused a favorable increment in the CA value of the trait.

#### Seed setting rate

On chromosome 10, only one SNP locus was found to be associated with the CA of seed-setting rate (Table 4, Supplementary Table 7). This SNP locus reduced the CA value by 19%. Three restorers, R4179, LC109 and Yanhui R8, contained this one locus in their genomes (Table 5).

## Discussion

### GBS assay for genome wide SNP discovery

The exploitation of heterosis in rice has increased the global rice-grain yield. This has led to an increase in productivity (growth, size, development, fertility and yield) of the progeny over that of both homozygous parents and is a fascinating phenomenon (Hochholdinger and Hoecker, 2007). The application of heterosis has been applied not only to rice but also to other crops such as tomatoes (Williams and Gilbert, 1960), maize (Shull, 1908), wheat (Singh et al., 2004), and soybeans (Pandini et al., 2002). In plants, heterosis is considered to be a complex trait that involves the presence of potential genetic distances, combining abilities and heterotic patterns of the parents traits (Beck et al., 1990). Rice breeders emphasized that heterosis in F_1_s is caused by the presence of combining ability alleles, that are present at different loci of crossing parents (Liu et al., 2002). They described how the combination of different alleles at a specific locus of crossing parents creates heterozygosity that results in the heterosis of offspring.

One part of our present study describes genome-wide SNP discovery in japonica rice. A large number of rice cultivars have been sequenced for SNP discovery to perform genetic and genomic experiments. However, only a few studies have been conducted on japonica rice (Nagasaki et al., 2010). Rice breeders have concluded that SNP discovery in japonica rice is challenging compared to that in indica rice, due to its close genetic relatedness and lower diversity (Glaszmann, 1987; Yang et al., 1994; Gao et al., 2005; Negrao et al., 2008). In our recent efforts, 18 parents of japonica rice were sequenced for SNP discovery, following optimized genotyping with a sequencing method. The use of two restriction enzymes based on a sequencing method of GBS is the most convenient, cost-effective and easy approach to the SNP discovery of large genome crop plants, including rice (Furuta et al., 2016). Nevertheless, this method also has the drawback of generating a large amount of missing data (Davey et al., 2011; Kim et al., 2016).

Here, the successful mapping of 60.4% of short reads onto the reference genome revealed 39,001 SNPs at 11,085 positions. The unique mapping of short reads plays a crucial role in the determination of SNPs (Nielsen et al., 2011). However, un-mapping reduced the chances of SNP discovery, which eventually occurs due to genomic deletions during the process of sequencing (Arai-Kichise et al., 2011). In our study, the number of SNPs among the parents varied. We observed higher polymorphisms in the nine restorer lines compared to the CMS lines. Of the 18 sequenced parents, the CMS Aizhixiang A reported a minimum number of SNPs. An in-depth analysis of the sequence genome of Aizhixiang A indicated that 70% of its sequence genome was similar to that of the Nipponbare reference. Furthermore, 25% of the missing data were also found, which further affected the chances for SNP discovery. We also found that the SNPs of CMS and the restorer lines were non-randomly distributed across 12 chromosomes and revealed several SNP-rich and SNP-poor intervals. Such extended SNP regions were similar to those reported in rice, wheat, mei (*Prunus mume* sieb. et zucc) and peach cultivars (Ravel et al., 2006; Subbaiyan et al., 2012; Fresnedo-Ramírez et al., 2013; Sun et al., 2013). In our study, there were also non-uniform patterns of SNPs within individual chromosomes, particularly at the terminal region of chromosome. We observed this situation at the terminal region of chromosome 11 for both the CMS and restorer lines. This may possibly arise due to the low variant rate at the centromere region of the chromosome, where recombination are typically more rare than at the chromosome terminal (Guo W. et al., 2007).

### Annotation of SNPs

In the sequenced genome of animals and plants, SNPs do not present equally throughout the genome. Some parts of the genome revealed more SNPs than others. The discovered SNPs in our study were annotated according to their positions in the genome. The annotation of identified SNPs indicates that more than half (57%) of the SNPs are within the intergenic region, whereas 24.4% of the SNPs have been found within the intron region of the genome. Only 20.4% of the SNPs occurred inside the exon regions, with 8.6% synonymous and 9.9% non-synonymous in nature. The number of SNPs in the exon region was 37% less than that in the intergenic regions. This is possibly, because of the higher frequency of polymorphisms evolving in intergenic spaces rather than coding sequences (Guo X. et al., 2007). However, the SNPs situated inside intergenic regions are also prominent, where there may have been the possibility of the presence of some functional alleles or genes (Salvi et al., 2007). In our study, the ratio of transitions to transversions was 1.4. This ratio was in line with former sequencing experiments in flax (Kumar et al., 2012).

### Evaluation of parents for GCA

The concept of combining ability (GCA and SCA) was developed by Sprague and Tatum in 1942. They characterized the breeding values of inbred lines by testing and evaluating them by combining ability evaluation trails (Sprague and Tatum, 1942). In every hybrid-breeding program, a breeder's goal is to select parents with good general combining ability and crosses that have high specific combining ability in their traits. From a genetic point of view, GCA evolved under additive and additive × additive gene action, whereas SCA evolved under non-additive gene action. The GCA of inbred parents is considered more important than the SCA, in which the parents were involved in random mating. The GCA evaluation of 18 parents of japonica hybrid rice indicated that most of the CMS lines exhibited maximum and positive GCA effect values for their studied traits. Previously, the same results of good general combining ability in the CMS lines were also reported (Singh et al., 1996). In contrast, most of the restorer lines also revealed greater GCA effect for their traits. The evidence of a higher GCA of restorer lines was also found in earlier studies (Rogbell et al., 1998).

### SNP-CA associations

With the discovery of genome-wide SNPs, several SNP-trait associations were reported in plant species, including maize, wheat, cotton, soybean, barley and *Arabidopsis thaliana* (Atwell et al., 2010; Yang et al., 2010; Pasam et al., 2012; Hu X. et al., 2015; Zhang et al., 2015; Su et al., 2016). In the past, most of the association studies on rice have focused on the improvement of yield-related traits (Huang et al., 2010; Zhao et al., 2011). Very few SNP associations with CA of traits have been performed. Here, we studied the heterozygous combinations of nucleotide bases (A-T/G-C) at a locus and observed its associations with the CA of traits. Moreover, we worked directly with the heterozygous association group and revealed 362 CA loci of 12 yield-related traits of japonica rice. For those results, each of the CMS and restorer lines contained a number of favorable or unfavorable SNP locus/loci of CA in the genome that caused a positive or negative effect on the traits. Few of the associated SNP loci were found to be co-associated with the CA of more than one yield-related trait. Among the CMS lines, 18A had a maximum number of negative SNP loci of CA for panicle and grain-related traits; whereas CMS A171 had a maximum number of positive SNP loci of CA for grain thickness and day to heading. For the restorer lines, LC64 and LR27 contained maximum numbers of positive SNP loci of CA for most of the studied traits. Previously, in maize, several favorable and unfavorable marker loci of CA were detected for five yield-contributing traits (Qi et al., 2013). Of these, the positive improved, while the negative decreased, the CA of the traits. Similarly, Qu et al. identified the CA loci of agronomical traits in rice by using QTL mapping of BCRILs (Qu et al., 2012). Moreover, all of the identified and associated SNP loci in our study were spread genome wide, with some on chromosomes 2, 5, 7, 9, and 11 exhibiting a maximum positive effect for the CA of traits.

The correct identification of SNPs based on annotation results made them informative. Previous studies have explained that the SNPs within the coding region can alter the promoter activities of gene expression, transcription and translation capability (LeVan et al., 2001; Alonso-Blanco et al., 2005; Ng and Henikoff, 2006; Shastry, 2009; Batley, 2015). Such identification and characterization of gene-based SNPs could be directly utilized in any breeding program regarding crop improvement (Wang et al., 2008; Huang et al., 2010). Among the 362 SNP loci detected in this study, 32 SNP loci for CA of seven traits were situated within the reported genes (Supplementary Table 8). For CA of plant height, we found 7 SNP loci within previously identified genes. These genes encode proteins for photosynthesis, hormonal activity, lipid binding and transporter activity. Of the seven reported genes for plant height, *Os03g0309200* encodes a proteins causing significant effect on photosynthesis activity, flowering time, and also have strong effects on stem and inter-node elongation in rice. Among four SNP loci for CA of grain width detected within the characterized genes, *Os06g0225300* is directly involved in improving rice architecture for high yield. Overall, the biological function of most of the reported genes were involved in photosynthesis, flower and embryo development, post-embryonic development, multi-cellular organismal development, cellular processes and metabolic processes. Similarly, the molecular function of these genes revealed DNA binding, lipid binding, nucleotide binding, protein binding, DNA binding transcription factor activity, catalytic activity and kinase activity. Such SNP loci situated inside the genes could be used as a candidate marker locus for tagging genomic regions responsible for improving CA of yield traits.

In conclusion, we present the identification of 215 positive and 147 negative coding-based SNP loci of CA for heterosis breeding in japonica rice. Our study implies that the identified SNP loci of CA will increase the selection efficiency of rice breeders in the proper selection of inbred parents. These insights into rice will be productive for future work to improve the CA of parents for yield-related traits and develop superior japonica hybrids by utilizing breeding designs. We expect that, the parental lines with more positive SNP loci of CA and less negative SNP loci of CA will be useful in hybrid breeding, because they have much higher potential for CA improvement for the traits.

## Author contributions

DH and IZ conceived the idea and designed the experiment; IZ, WT, EL, SK, HW, and EM contributed to the data collection; IZ analyzed the data and wrote the paper.

## Funding

Funding support was provided by a grant from the National Natural Science Foundation of China (31571743 and 31671658), Chinese national “863” program (2010AA101301), a special program of scientific research belonging to the Educational Ministry of China (KYZ2012-9) and a grant from the doctoral fund of the Educational Ministry of China (20130097110001), a grant from the doctoral fund of the Educational Ministry of China (20130097110001).

### Conflict of interest statement

The authors declare that the research was conducted in the absence of any commercial or financial relationships that could be construed as a potential conflict of interest.
