# Supplementary material for: Genome-Wide Single-Nucleotide Polymorphisms in CMS and Restorer Lines Discovered by Genotyping Using Sequencing and Association with Marker-Combining Ability for 12 Yield-Related Traits in Oryza sativa L. subsp. Japonica
Source: Front Plant Sci. 2017 Feb 8;8:143. doi: 10.3389/fpls.2017.00143 (PMC5297617; doi:10.3389/fpls.2017.00143)
Supplement: Supplementary file 1 [file Table1.DOCX]

**Supplementary Table 1 (a)** SNPs in genomic DNA observed in CMS 95122A in comparison with Nipponbare reference genome.

| **Chromosome** | **Length** | **Variants** | **Variants rate** |
| --- | --- | --- | --- |
| 1 | 43,270,923 | 233 | 185,712 |
| 2 | 35,937,250 | 116 | 309,803 |
| 3 | 36,413,819 | 53 | 687,053 |
| 4 | 35,502,694 | 84 | 422,651 |
| 5 | 29,958,434 | 47 | 637,413 |
| 6 | 31,248,787 | 53 | 589,599 |
| 7 | 29,697,621 | 49 | 606,073 |
| 8 | 28,443,022 | 73 | 389,630 |
| 9 | 23,012,720 | 81 | 284,107 |
| 10 | 23,207,287 | 39 | 595,058 |
| 11 | 29,021,106 | 248 | 117,020 |
| 12 | 27,531,856 | 302 | 91,165 |
| **Total** | **373,245,519** | **1,378** | **270,860** |

**Supplementary Table 1 (b)** SNPs in genomic DNA observed in CMS 90167A in comparison with Nipponbare reference genome.

| **Chromosome** | **Length** | **Variants** | **Variants rate** |
| --- | --- | --- | --- |
| 1 | 43,270,923 | 153 | 282,816 |
| 2 | 35,937,250 | 178 | 201,894 |
| 3 | 36,413,819 | 91 | 400,151 |
| 4 | 35,502,694 | 77 | 461,073 |
| 5 | 29,958,434 | 16 | 1,872,402 |
| 6 | 31,248,787 | 109 | 286,686 |
| 7 | 29,697,621 | 113 | 262,810 |
| 8 | 28,443,022 | 55 | 517,145 |
| 9 | 23,012,720 | 130 | 177,020 |
| 10 | 23,207,287 | 38 | 610,718 |
| 11 | 29,021,106 | 235 | 123,494 |
| 12 | 27,531,856 | 110 | 250,289 |
| **Total** | **373,245,519** | **1,305** | **286,011** |

**Supplementary Table 1 (c)** SNPs in genomic DNA observed CMS 863A in comparison with Nipponbare reference genome.

| **Chromosome** | **Length** | **Variants** | **Variants rate** |
| --- | --- | --- | --- |
| 1 | 43,270,923 | 284 | 152,362 |
| 2 | 35,937,250 | 65 | 552,880 |
| 3 | 36,413,819 | 86 | 423,416 |
| 4 | 35,502,694 | 56 | 633,976 |
| 5 | 29,958,434 | 8 | 3,744,804 |
| 6 | 31,248,787 | 45 | 694,417 |
| 7 | 29,697,621 | 55 | 539,956 |
| 8 | 28,443,022 | 65 | 437,584 |
| 9 | 23,012,720 | 23 | 1,000,553 |
| 10 | 23,207,287 | 166 | 139,802 |
| 11 | 29,021,106 | 364 | 79,728 |
| 12 | 27,531,856 | 56 | 491,640 |
| **Total** | **373,245,519** | **1,273** | **293,201** |

**Supplementary Table 1 (d)** SNPs in genomic DNA observed in CMS A171 in comparison with Nipponbare reference genome.

| **Chromosome** | **Length** | **Variants** | **Variants rate** |
| --- | --- | --- | --- |
| 1 | 43,270,923 | 147 | 294,360 |
| 2 | 35,937,250 | 129 | 278,583 |
| 3 | 36,413,819 | 83 | 438,720 |
| 4 | 35,502,694 | 53 | 669,862 |
| 5 | 29,958,434 | 25 | 1,198,337 |
| 6 | 31,248,787 | 78 | 400,625 |
| 7 | 29,697,621 | 84 | 353,543 |
| 8 | 28,443,022 | 58 | 490,396 |
| 9 | 23,012,720 | 93 | 247,448 |
| 10 | 23,207,287 | 33 | 703,251 |
| 11 | 29,021,106 | 297 | 97,714 |
| 12 | 27,531,856 | 126 | 218,506 |
| **Total** | **373,245,519** | **1,206** | **309,490** |

**Supplementary Table 1 (e)** SNPs in genomic DNA observed in CMS Aizhixiang A in comparison with Nipponbare reference genome.

| **Chromosome** | **Length** | **Variants** | **Variants rate** |
| --- | --- | --- | --- |
| 1 | 43,270,923 | 15 | 2,884,728 |
| 2 | 35,937,250 | 12 | 2,994,770 |
| 3 | 36,413,819 | 14 | 2,600,987 |
| 4 | 35,502,694 | 7 | 5,071,813 |
| 5 | 29,958,434 | 7 | 4,279,776 |
| 6 | 31,248,787 | 1 | 31,248,787 |
| 7 | 29,697,621 | 53 | 560,332 |
| 8 | 28,443,022 | 2 | 14,221,511 |
| 9 | 23,012,720 | 43 | 535,179 |
| 10 | 23,207,287 | 10 | 2,320,728 |
| 11 | 29,021,106 | 178 | 163,039 |
| 12 | 27,531,856 | 23 | 1,197,037 |
| **Total** | **373,245,519** | **365** | **1,022,590** |

**Supplementary Table 1 (f)** SNPs in genomic DNA observed in CMS 18A in comparison with Nipponbare reference genome.

| **Chromosome** | **Length** | **Variants** | **Variants rate** |
| --- | --- | --- | --- |
| 1 | 43,270,923 | 113 | 382,928 |
| 2 | 35,937,250 | 43 | 835,750 |
| 3 | 36,413,819 | 52 | 700,265 |
| 4 | 35,502,694 | 58 | 612,115 |
| 5 | 29,958,434 | 32 | 936,201 |
| 6 | 31,248,787 | 31 | 1,008,025 |
| 7 | 29,697,621 | 92 | 322,800 |
| 8 | 28,443,022 | 43 | 661,465 |
| 9 | 23,012,720 | 62 | 371,172 |
| 10 | 23,207,287 | 26 | 892,587 |
| 11 | 29,021,106 | 249 | 116,550 |
| 12 | 27,531,856 | 87 | 316,458 |
| **Total** | **373,245,519** | **888** | **420,321** |

**Supplementary Table 1 (g)** SNPs in genomic DNA observed in CMS Zhe 04A in comparison with Nipponbare reference genome.

| **Chromosome** | **Length** | **Variants** | **Variants rate** |
| --- | --- | --- | --- |
| 1 | 43,270,923 | 143 | 302,593 |
| 2 | 35,937,250 | 131 | 274,330 |
| 3 | 36,413,819 | 97 | 375,400 |
| 4 | 35,502,694 | 99 | 358,613 |
| 5 | 29,958,434 | 15 | 1,997,228 |
| 6 | 31,248,787 | 123 | 254,055 |
| 7 | 29,697,621 | 122 | 243,423 |
| 8 | 28,443,022 | 97 | 293,227 |
| 9 | 23,012,720 | 110 | 209,206 |
| 10 | 23,207,287 | 111 | 209,074 |
| 11 | 29,021,106 | 367 | 79,076 |
| 12 | 27,531,856 | 370 | 74,410 |

**Supplementary Table 1 (h)** SNPs in genomic DNA observed CMS Chunjiang 19A in comparison with Nipponbare reference genome.

| **Chromosome** | **Length** | **Variants** | **Variants rate** |
| --- | --- | --- | --- |
| 1 | 43,270,923 | 166 | 260,668 |
| 2 | 35,937,250 | 75 | 479,163 |
| 3 | 36,413,819 | 423 | 86,084 |
| 4 | 35,502,694 | 24 | 1,479,278 |
| 5 | 29,958,434 | 68 | 440,565 |
| 6 | 31,248,787 | 185 | 168,912 |
| 7 | 29,697,621 | 48 | 618,700 |
| 8 | 28,443,022 | 134 | 212,261 |
| 9 | 23,012,720 | 47 | 489,632 |
| 10 | 23,207,287 | 43 | 539,704 |
| 11 | 29,021,106 | 313 | 92,719 |
| 12 | 27,531,856 | 48 | 573,580 |
| **Total** | **373,245,519** | **1,574** | **237,131** |

**Supplementary Table 1 (i)** SNPs in genomic DNA observed in CMS Chunjiang 18A in comparison with Nipponbare reference genome.

| **Chromosome** | **Length** | **Variants** | **Variants rate** |
| --- | --- | --- | --- |
| 1 | 43,270,923 | 200 | 216,354 |
| 2 | 35,937,250 | 160 | 224,607 |
| 3 | 36,413,819 | 489 | 74,465 |
| 4 | 35,502,694 | 261 | 136,025 |
| 5 | 29,958,434 | 153 | 195,806 |
| 6 | 31,248,787 | 466 | 67,057 |
| 7 | 29,697,621 | 128 | 232,012 |
| 8 | 28,443,022 | 160 | 177,768 |
| 9 | 23,012,720 | 107 | 215,072 |
| 10 | 23,207,287 | 358 | 64,824 |
| 11 | 29,021,106 | 489 | 59,347 |
| 12 | 27,531,856 | 441 | 62,430 |
| **Total** | **373,245,519** | **3,412** | **109,392** |
