# Supplementary material for: Genome-Wide Single-Nucleotide Polymorphisms in CMS and Restorer Lines Discovered by Genotyping Using Sequencing and Association with Marker-Combining Ability for 12 Yield-Related Traits in Oryza sativa L. subsp. Japonica
Source: Front Plant Sci. 2017 Feb 8;8:143. doi: 10.3389/fpls.2017.00143 (PMC5297617; doi:10.3389/fpls.2017.00143)
Supplement: Supplementary file 2 [file Table2.DOCX]

**Supplementary Table 2 (a)** SNPs in genomic DNA observed in restorer R4179 in comparison with Nipponbare reference genome.

| **Chromosome** | **Length** | **Variants** | **Variants rate** |
| --- | --- | --- | --- |
| 1 | 43,270,923 | 41 | 1,055,388 |
| 2 | 35,937,250 | 424 | 84,757 |
| 3 | 36,413,819 | 120 | 303,448 |
| 4 | 35,502,694 | 38 | 934,281 |
| 5 | 29,958,434 | 67 | 447,140 |
| 6 | 31,248,787 | 71 | 440,123 |
| 7 | 29,697,621 | 85 | 349,383 |
| 8 | 28,443,022 | 138 | 206,108 |
| 9 | 23,012,720 | 107 | 215,072 |
| 10 | 23,207,287 | 179 | 129,649 |
| 11 | 29,021,106 | 183 | 158,585 |
| 12 | 27,531,856 | 55 | 500,579 |
| **Total** | **373,245,519** | **1,508** | **247,510** |

**Supplementary Table 2 (b)** SNPs in genomic DNA observed in restorer LC64 in comparison with Nipponbare reference genome.

| **Chromosome** | **Length** | **Variants** | **Variants rate** |
| --- | --- | --- | --- |
| 1 | 43,270,923 | 361 | 119,864 |
| 2 | 35,937,250 | 237 | 151,633 |
| 3 | 36,413,819 | 159 | 229,017 |
| 4 | 35,502,694 | 118 | 300,870 |
| 5 | 29,958,434 | 275 | 108,939 |
| 6 | 31,248,787 | 238 | 131,297 |
| 7 | 29,697,621 | 95 | 312,606 |
| 8 | 28,443,022 | 307 | 92,648 |
| 9 | 23,012,720 | 42 | 547,921 |
| 10 | 23,207,287 | 176 | 131,859 |
| 11 | 29,021,106 | 658 | 44,105 |
| 12 | 27,531,856 | 304 | 90,565 |
| **Total** | **373,245,519** | **2,970** | **125,671** |

**Supplementary Table 2 (c)** SNPs in genomic DNA observed in restorer LC109 in comparison with Nipponbare reference genome

| **Chromosome** | **Length** | **Variants** | **Variants rate** |
| --- | --- | --- | --- |
| 1 | 43,270,923 | 103 | 420,106 |
| 2 | 35,937,250 | 41 | 876,518 |
| 3 | 36,413,819 | 78 | 466,843 |
| 4 | 35,502,694 | 58 | 612,115 |
| 5 | 29,958,434 | 11 | 2,723,494 |
| 6 | 31,248,787 | 363 | 86,084 |
| 7 | 29,697,621 | 60 | 494,960 |
| 8 | 28,443,022 | 219 | 129,876 |
| 9 | 23,012,720 | 42 | 547,921 |
| 10 | 23,207,287 | 75 | 309,430 |
| 11 | 29,021,106 | 364 | 79,728 |
| 12 | 27,531,856 | 113 | 243,644 |
| **Total** | **373,245,519** | **1,527** | **244,430** |

**Supplementary Table 2 (d)** SNPs in genomic DNA observed in restorer Yanhui R50 in comparison with Nipponbare reference genome.

| **Chromosome** | **Length** | **Variants** | **Variants rate** |
| --- | --- | --- | --- |
| 1 | 43,270,923 | 232 | 186,512 |
| 2 | 35,937,250 | 512 | 70,189 |
| 3 | 36,413,819 | 64 | 568,965 |
| 4 | 35,502,694 | 45 | 788,948 |
| 5 | 29,958,434 | 204 | 146,855 |
| 6 | 31,248,787 | 229 | 136,457 |
| 7 | 29,697,621 | 471 | 63,052 |
| 8 | 28,443,022 | 190 | 149,700 |
| 9 | 23,012,720 | 78 | 295,034 |
| 10 | 23,207,287 | 104 | 223,146 |
| 11 | 29,021,106 | 440 | 65,957 |
| 12 | 27,531,856 | 54 | 509,849 |
| **Total** | **373,245,519** | **2,623** | **142,297** |

**Supplementary Table 2 (e)** SNPs in genomic DNA observed in restorer Yanhui R8 in comparison with Nipponbare reference genome.

| **Chromosome** | **Length** | **Variants** | **Variants rate** |
| --- | --- | --- | --- |
| 1 | 43,270,923 | 224 | 193,173 |
| 2 | 35,937,250 | 340 | 105,697 |
| 3 | 36,413,819 | 83 | 438,720 |
| 4 | 35,502,694 | 36 | 986,185 |
| 5 | 29,958,434 | 292 | 102,597 |
| 6 | 31,248,787 | 272 | 114,885 |
| 7 | 29,697,621 | 708 | 41,945 |
| 8 | 28,443,022 | 322 | 88,332 |
| 9 | 23,012,720 | 105 | 219,168 |
| 10 | 23,207,287 | 202 | 114,887 |
| 11 | 29,021,106 | 797 | 36,412 |
| 12 | 27,531,856 | 102 | 269,920 |
| **Total** | **373,245,519** | **3,483** | **107,162** |

**Supplementary Table 2 (f)** SNPs in genomic DNA observed in restorer LR5 in comparison with Nipponbare reference genome.

| **Chromosome** | **Length** | **Variants** | **Variants rate** |
| --- | --- | --- | --- |
| 1 | 43,270,923 | 453 | 95,520 |
| 2 | 35,937,250 | 933 | 38,517 |
| 3 | 36,413,819 | 702 | 51,871 |
| 4 | 35,502,694 | 53 | 669,862 |
| 5 | 29,958,434 | 313 | 95,713 |
| 6 | 31,248,787 | 398 | 78,514 |
| 7 | 29,697,621 | 566 | 52,469 |
| 8 | 28,443,022 | 351 | 81,034 |
| 9 | 23,012,720 | 92 | 250,138 |
| 10 | 23,207,287 | 514 | 45,150 |
| 11 | 29,021,106 | 945 | 30,710 |
| 12 | 27,531,856 | 77 | 357,556 |
| **Total** | **373,245,519** | **5,397** | **69,157** |

**Supplementary Table 2 (g)** SNPs in genomic DNA observed in restorer LR 27 in comparison with Nipponbare reference genome.

| **Chromosome** | **Length** | **Variants** | **Variants rate** |
| --- | --- | --- | --- |
| 1 | 43,270,923 | 499 | 86,715 |
| 2 | 35,937,250 | 563 | 63,831 |
| 3 | 36,413,819 | 543 | 67,060 |
| 4 | 35,502,694 | 33 | 1,075,839 |
| 5 | 29,958,434 | 134 | 223,570 |
| 6 | 31,248,787 | 409 | 76,402 |
| 7 | 29,697,621 | 534 | 55,613 |
| 8 | 28,443,022 | 300 | 94,810 |
| 9 | 23,012,720 | 132 | 174,338 |
| 10 | 23,207,287 | 370 | 62,722 |
| 11 | 29,021,106 | 527 | 55,068 |
| 12 | 27,531,856 | 84 | 327,760 |

**Supplementary Table 2 (h)** SNPs in genomic DNA observed in restorer Shenhui254 in comparison with Nipponbare reference genome.

| **Chromosome** | **Length** | **Variants** | **Variants rate** |
| --- | --- | --- | --- |
| 1 | 43,270,923 | 224 | 193,173 |
| 2 | 35,937,250 | 269 | 133,595 |
| 3 | 36,413,819 | 37 | 984,157 |
| 4 | 35,502,694 | 51 | 696,131 |
| 5 | 29,958,434 | 256 | 117,025 |
| 6 | 31,248,787 | 204 | 153,180 |
| 7 | 29,697,621 | 190 | 156,303 |
| 8 | 28,443,022 | 70 | 406,328 |
| 9 | 23,012,720 | 109 | 211,125 |
| 10 | 23,207,287 | 109 | 212,910 |
| 11 | 29,021,106 | 552 | 52,574 |
| 12 | 27,531,856 | 74 | 372,052 |
| **Total** | **373,245,519** | **2,145** | **174,007** |

**Supplementary Table 2 (i)** SNPs in genomic DNA observed in restorer C4115 in comparison with Nipponbare reference genome.

| **Chromosome** | **Length** | **Variants** | **Variants rate** |
| --- | --- | --- | --- |
| 1 | 43,270,923 | 229 | 188,955 |
| 2 | 35,937,250 | 362 | 99,274 |
| 3 | 36,413,819 | 264 | 137,931 |
| 4 | 35,502,694 | 32 | 1,109,459 |
| 5 | 29,958,434 | 100 | 299,584 |
| 6 | 31,248,787 | 141 | 221,622 |
| 7 | 29,697,621 | 102 | 291,153 |
| 8 | 28,443,022 | 125 | 227,544 |
| 9 | 23,012,720 | 76 | 302,798 |
| 10 | 23,207,287 | 165 | 140,650 |
| 11 | 29,021,106 | 379 | 76,572 |
| 12 | 27,531,856 | 59 | 466,641 |
| **Total** | **373,245,519** | **2,034** | **183,503** |
