# Supplementary material for: Genome-Wide Single-Nucleotide Polymorphisms in CMS and Restorer Lines Discovered by Genotyping Using Sequencing and Association with Marker-Combining Ability for 12 Yield-Related Traits in Oryza sativa L. subsp. Japonica
Source: Front Plant Sci. 2017 Feb 8;8:143. doi: 10.3389/fpls.2017.00143 (PMC5297617; doi:10.3389/fpls.2017.00143)
Supplement: Supplementary file 3 [file Table3.DOCX]

**Supplementary Table 3** Detail annotation of identified SNPs of nine CMS lines based on Nipponbare reference sequence

| REGIONS | **95122A** | **90167A** | **863A** | **A171** | **Aizhixiang A** | **18A** | **Zhe 04A** | **Chunjiang 19A** | **Chunjiang 18A** |
| --- | --- | --- | --- | --- | --- | --- | --- | --- | --- |
| **3_prime_UTR_variant** | 107 | 81 | 108 | 79 | 20 | 53 | 128 | 116 | 244 |
| **5_prime_UTR_premature_start_codon_gain_variant** | 4 | 6 | 8 | 3 | 5 | 6 | 7 | 7 | 14 |
| **5_prime_UTR_variant** | 48 | 30 | 53 | 44 | 11 | 40 | 65 | 54 | 109 |
| **downstream_gene_variant** | 2,279 | 2,113 | 2,239 | 2,226 | 614 | 1,420 | 2,810 | 2,468 | 5,238 |
| **intergenic_region** | 793 | 770 | 700 | 702 | 219 | 516 | 1,026 | 884 | 1,972 |
| **intron_variant** | 357 | 328 | 271 | 277 | 84 | 213 | 399 | 430 | 853 |
| **missense_variant** | 137 | 109 | 132 | 115 | 29 | 73 | 156 | 147 | 304 |
| **non_coding_exon_variant** | 22 | 33 | 43 | 22 | 7 | 16 | 33 | 24 | 74 |
| **splice_region_variant** | 1 | 2 | 2 | 2 | - | 1 | 3 | 1 | 3 |
| **splice_region_variant+intron_variant** | 8 | 15 | 12 | 7 | 1 | 2 | 13 | 10 | 32 |
| **splice_region_variant+non_coding_exon_variant** | 1 | 1 | 4 | 1 | - | 21 | 2 | 3 | 5 |
| **splice_region_variant+synonymous_variant** | 2 | 1 | - | 2 | - | 1 | 2 | 4 | 5 |
| **stop_gained** | - | 2 | 3 | 3 | 1 | 2 | 6 | 1 | 8 |
| **stop_lost** | 2 | 1 | - | 1 | - | 2 | 2 | - | 2 |
| **synonymous_variant** | 117 | 119 | 129 | 117 | 30 | 75 | 164 | 112 | 264 |
| **upstream_gene_variant** | 1960 | 1,871 | 1942 | 1,800 | 523 | 1,220 | 2,353 | 2,244 | 4,938 |
