# Supplementary material for: Genome-Wide Single-Nucleotide Polymorphisms in CMS and Restorer Lines Discovered by Genotyping Using Sequencing and Association with Marker-Combining Ability for 12 Yield-Related Traits in Oryza sativa L. subsp. Japonica
Source: Front Plant Sci. 2017 Feb 8;8:143. doi: 10.3389/fpls.2017.00143 (PMC5297617; doi:10.3389/fpls.2017.00143)
Supplement: Supplementary file 4 [file Table4.DOCX]

**Supplementary Table 4** Detail annotations of identified SNPs of nine restorer lines based on Nipponbare reference sequence

| REGIONS | **R4179** | **LC64** | **LC109** | **Yanhui R50** | **Yanhui R8** | **LR5** | **LR27** | **Shenhui254** | **C4115** |
| --- | --- | --- | --- | --- | --- | --- | --- | --- | --- |
| **3_prime_UTR_variant** | 98 | 191 | 90 | 179 | 205 | 367 | 286 | 104 | 151 |
| **5_prime_UTR_premature_start_codon_gain_variant** | 11 | 18 | 4 | 13 | 14 | 26 | 22 | 9 | 12 |
| **5_prime_UTR_variant** | 46 | 118 | 49 | 97 | 93 | 179 | 140 | 58 | 61 |
| **downstream_gene_variant** | 2,589 | 4,845 | 2,540 | 4,238 | 5,277 | 8,553 | 6,370 | 3,230 | 3,169 |
| **intergenic_region** | 850 | 1,774 | 904 | 1,516 | 2,057 | 3,080 | 2,364 | 1,288 | 1,137 |
| **intron_variant** | 342 | 639 | 338 | 604 | 856 | 1,372 | 1,058 | 479 | 538 |
| **missense_variant** | 171 | 299 | 136 | 264 | 327 | 518 | 417 | 202 | 215 |
| **non_coding_exon_variant** | 1 | 1 | 32 | 64 | 72 | 150 | 95 | 53 | 56 |
| **splice_region_variant** | 40 | 3 | 1 | 3 | 3 | 4 | 3 | - | - |
| **splice_region_variant+intron_variant** | 12 | 28 | 11 | 19 | 28 | 45 | 26 | 19 | 19 |
| **splice_region_variant+non_coding_exon_variant** | 2 | - | - | 1 | 1 | 7 | 8 | 1 | 1 |
| **splice_region_variant+synonymous_variant** | 2 | 4 | - | 2 | 3 | 4 | 5 | 1 |  |
| **stop_gained** | 1 | 3 | 1 | 3 | 4 | 4 | 1 | 2 | 1 |
| **stop_lost** | 1 | 1 |  | 1 | 2 | 1 | 1 | 4 | 1 |
| **synonymous_variant** | 140 | 272 | 130 | 203 | 288 | 435 | 313 | 194 | 191 |
| **upstream_gene_variant** | 2,315 | 4,372 | 2250 | 3,789 | 4883 | 7712 | 5,905 | 2,968 | 2,997 |
