# Supplementary material for: Genome-Wide Single-Nucleotide Polymorphisms in CMS and Restorer Lines Discovered by Genotyping Using Sequencing and Association with Marker-Combining Ability for 12 Yield-Related Traits in Oryza sativa L. subsp. Japonica
Source: Front Plant Sci. 2017 Feb 8;8:143. doi: 10.3389/fpls.2017.00143 (PMC5297617; doi:10.3389/fpls.2017.00143)
Supplement: Supplementary file 5 [file Table5.DOCX]

**Supplementary Table 5** Detail classifications of base substitutions of SNPs identified in the nine CMS lines.

| Substitutions | **95122A** | **90167A** | **863A** | **A171** | **Aizhixiang A** | **18A** | **Zhe 04A** | **Chunjiang 19A** | **Chunjiang 18A** |
| --- | --- | --- | --- | --- | --- | --- | --- | --- | --- |
| Transitions (Ts) |  |  |  |  |  |  |  |  |  |
| C/T | 216 | 185 | 204 | 163 | 58 | 515 | 270 | 235 | 515 |
| G/A | 212 | 194 | 194 | 180 | 60 | 537 | 268 | 243 | 537 |
| Transversions (Tv) |  |  |  |  |  |  |  |  |  |
| C/G | 50 | 52 | 48 | 41 | 13 | 119 | 68 | 45 | 119 |
| T/A | 88 | 70 | 69 | 70 | 22 | 195 | 94 | 107 | 195 |
| A/C | 64 | 65 | 64 | 72 | 16 | 193 | 103 | 98 | 193 |
| G/T | 60 | 67 | 55 | 66 | 20 | 151 | 85 | 82 | 151 |
| Ts/Tv ratio | 1.4 | 1.3 | 1.5 | 1.2 | 1.5 | 1.5 | 1.4 | 1.3 | 1.5 |
