# Supplementary material for: Genome-Wide Single-Nucleotide Polymorphisms in CMS and Restorer Lines Discovered by Genotyping Using Sequencing and Association with Marker-Combining Ability for 12 Yield-Related Traits in Oryza sativa L. subsp. Japonica
Source: Front Plant Sci. 2017 Feb 8;8:143. doi: 10.3389/fpls.2017.00143 (PMC5297617; doi:10.3389/fpls.2017.00143)
Supplement: Supplementary file 6 [file Table6.DOCX]

**Supplementary Table 6** Detail classifications of base substitutions of SNPs identified in the nine restorer lines

| Substitutions | **R4179** | **LC64** | **LC109** | **Yanhui R50** | **Yanhui R8** | **LR5** | **LR27** | **Shenhui254** | **C4115** |
| --- | --- | --- | --- | --- | --- | --- | --- | --- | --- |
| Transitions (Ts) |  |  |  |  |  |  |  |  |  |
| C/T | 235 | 468 | 226 | 393 | 542 | 834 | 649 | 316 | 309 |
| G/A | 453 | 453 | 208 | 404 | 511 | 896 | 650 | 313 | 310 |
| Transversions (Tv) |  |  |  |  |  |  |  |  |  |
| C/G | 56 | 114 | 66 | 104 | 136 | 191 | 142 | 77 | 74 |
| T/A | 91 | 175 | 102 | 163 | 306 | 306 | 219 | 124 | 111 |
| A/C | 89 | 135 | 205 | 117 | 250 | 250 | 204 | 118 | 101 |
| G/T | 86 | 159 | 226 | 147 | 182 | 278 | 213 | 123 | 115 |
| Ts/Tv ratio | 1.4 | 1.4 | 1.3 | 1.4 | 1.5 | 1.5 | 1.5 | 1.4 | 1.4 |
