# Supplementary material for: Genome-Wide Single-Nucleotide Polymorphisms in CMS and Restorer Lines Discovered by Genotyping Using Sequencing and Association with Marker-Combining Ability for 12 Yield-Related Traits in Oryza sativa L. subsp. Japonica
Source: Front Plant Sci. 2017 Feb 8;8:143. doi: 10.3389/fpls.2017.00143 (PMC5297617; doi:10.3389/fpls.2017.00143)
Supplement: Supplementary file 8 [file Table8.DOCX]

**Supplementary Table 8** list of associated SNPs of CA for seven traits situated inside of already reported genes

| **Traits** | **SNP Position** | **Chr** | **Gene ID** | **Biological- function** | **Molecular -function** | **References** |
| --- | --- | --- | --- | --- | --- | --- |
| **Plant height** | 10471299 | 1 | Os01g0289600 | nucleobase, nucleoside, nucleotide and nucleic acid metabolic process | sequence-specific DNA binding transcription factor activity | ([Ross et al., 2007](#_ENREF_31)), ([Xie and Shen, 2005](#_ENREF_38)) |
|  | 10685754 | 3 | Os03g0302900 | mitochondrion | Mitochondrion (cellular compenent) | ([Asano et al., 2009](#_ENREF_1)),([Asano et al.](#_ENREF_2)) |
|  | 11022164 | 3 | Os03g0309200 | photosynthesis | signal transduceractivity | ([Liu et al., 2012](#_ENREF_23)), ([Huang et al., 2012b](#_ENREF_12)), ([Ishikawa et al., 2011](#_ENREF_15)), ([Iwamoto et al., 2011](#_ENREF_17)) ,  ([Lee et al., 2010](#_ENREF_20)), ([Takano et al., 2009](#_ENREF_34)), ([Takano and Shinomura, 2005](#_ENREF_35)) |
|  | 35337258 | 4 | Os04g0690800 | response to abiotic stimulus | lipid binding | ([Ishida et al., 2011](#_ENREF_14)) |
|  | 29073164 | 6 | Os06g0695800 | transport | transporter activity | ([Huang et al., 2009](#_ENREF_10)) |
|  | 788571 | 11 | Os11g0117500 | nucleobase, nucleoside, nucleotide and nucleic acid metabolic process | sequence-specific DNA binding transcription factor activity | ([Ross et al., 2007](#_ENREF_31)), ([Xie and Shen, 2005](#_ENREF_38)) |
|  | 16500692 | 12 | Os12g0465700 | Plant-specific protein containing a glutamine-rich region and a conserved motif, Controls of phyllotaxy by affecting cytokinin signaling | Plant-specific protein containing a glutamine-rich region and a conserved motif, Controls of phyllotaxy by affecting cytokinin signaling | ([Itoh et al., 2012](#_ENREF_16)) |
| **Grain width** | 878185 | 5 | Os05g0116100 | metabolic process | catalytic activity | ([Kim et al., 2013](#_ENREF_18)) |
|  | 6486229 | 6 | Os06g0225300 | flower development, embryo development | kinase activity | ([Li et al., 2009](#_ENREF_22)) |
|  | 29627879 | 7 | Os07g0695100 | \|  \| nucleobase, nucleoside, nucleotide and nucleic acid metabolic process \| \| --- \| --- \| | DNA binding | ([Gao et al., 2014](#_ENREF_6)), ([Liu et al., 2013](#_ENREF_24)), ([Yan et al., 2013](#_ENREF_39)), ([Murakami et al., 2005](#_ENREF_26)), |
|  | 18367541 | 10 | Os10g0485600 |  |  | ([Meenu Kapoor, 2008](#_ENREF_25)) |
| **Grain length** | 1157951 | 5 | Os05g0121600 | post-embryonic development | sequence-specific DNA binding transcription factor activity | ([Fu and Xue, 2010](#_ENREF_5)) |
| **Grain thickness** | 4822199 | 1 | Os01g0190400 | nucleobase, nucleoside, nucleotide and nucleic acid metabolic process | sequence-specific DNA binding transcription factor activity | ([Fu and Xue, 2010](#_ENREF_5)) |
| **1000-grain weight** | 32831926 | 2 | Os02g0776700 | nucleobase, nucleoside, nucleotide and nucleic acid metabolic process | sequence-specific DNA binding transcription factor activity | ([Washio, 2015](#_ENREF_37)) |
| **Panicle length** | 40906379 | 1 | Os01g0932500 | transport | transporter activity | ([Bañuelos et al., 2002](#_ENREF_3)) |
|  | 7711988 | 2 | Os02g0236100 | signal transduction | nucleotide binding | ([Huang et al., 2012a](#_ENREF_11)) |
|  | 4924144 | 3 | Os02g0194900 | nucleobase, nucleoside, nucleotide and nucleic acid metabolic | \| sequence-specific DNA binding transcription factor activity \|  \| \| --- \| --- \| | ([Nijhawan et al., 2008](#_ENREF_28)) |
|  | 4999202 | 3 | Os02g0196000 | transport | transporter activity | ([Chen et al., 2008](#_ENREF_4)) |
|  | 34431183 | 3 | Os03g0820500 | response to stress | protein binding | ([Huang et al., 2012c](#_ENREF_13)) |
|  | 34685369 | 3 | Os03g0825700 | anatomical structure morphogenesis | cellular process | ([Yu et al., 2016](#_ENREF_40)) |
|  | 35337250 | 4 | Os04g0690800 | response to abiotic stimulus | lipid binding | ([Ishida et al., 2011](#_ENREF_14)) |
|  | 24723022 | 7 | Os07g0603800 | transport | transporter activity | ([Léran et al., 2013](#_ENREF_21)) |
|  | 19099683 | 10 | Os10g0500700 | cellular process | molecular_function | ([Garg et al., 2010](#_ENREF_7)) |
|  | 23107589 | 10 | Os10g0579400 | multicellular organismal development | sequence-specific DNA binding transcription factor activity | ([Xie and Shen, 2005](#_ENREF_38)) |
|  | 16500770 | 12 | Os12g0465700 | biological_process | molecular_function | ([Itoh et al., 2012](#_ENREF_16)) |
| **Panicles number per plant** | 28036316 | 5 | Os05g0563400 | response to endogenous stimulus | DNA binding | ([Wang and Al., 2007](#_ENREF_36)), ([Sato et al., 2002](#_ENREF_32)) |
|  | 5560591 | 1 | Os01g0201700 | flower development | DNA binding | ([Hu et al., 2015](#_ENREF_9)) |
|  | 6444474 | 1 | Os01g0217900 | regulation of gene expression, epigenetic | catalytic activity | ([Ono et al., 2012](#_ENREF_29)) |
|  | 24571861 | 2 | Os02g0618200 | biological_process | protein binding | ([Nakamura et al., 2007](#_ENREF_27)) |
|  | 35337258 | 4 | Os04g0690800 | response to abiotic stimulus | lipid binding | ([Ishida et al., 2011](#_ENREF_14)) |
|  | 19099683 | 10 | Os10g0500700 | metabolic process | cytosol | ([Garg et al., 2010](#_ENREF_7)) |
|  | 16500692 | 12 | Os12g0465700 | biological_process | molecular_function | ([Itoh et al., 2012](#_ENREF_16)) |
